# Migration patterns of uncemented femoral stems in hip replacement: a systematic review and meta-analysis of clinical radiostereometric analysis cohort studies

**DOI:** 10.2340/17453674.2026.45412

**Published:** 2026-04-16

**Authors:** Lisa VAN DER WATER, Christiaan H RIGHOLT, Trevor GASCOYNE, Bart L KAPTEIN, Rob G H H NELISSEN, Bart G C W PIJLS

**Affiliations:** 1 Department of Orthopaedics, Leiden University Medical Center, Leiden, The Netherlands; 2 Orthopaedic Innovation Centre, Winnipeg, MB, Canada; 3 Department of Surgery, University of Manitoba, Winnipeg, MB, Canada; 4 Biomedical Engineering Program, University of Manitoba, Winnipeg, MB, Canada

## Abstract

**Background and purpose:**

We conducted a systematic review and meta-analysis of radiostereometric analysis studies of primary uncemented femoral stems to investigate their subsidence and retroversion patterns and the migration patterns according to implant, patient, and study characteristics.

**Methods:**

A systematic search of PubMed, Web of Science, Cochrane, and Embase databases to identify all radiostereometric analysis studies on femoral stem migration following primary uncemented total hip replacement was performed. Clinical studies with 2 or more postoperative radiostereometric measurements within 2 years were included. Subsidence and retroversion at 6 weeks, 3 and 6 months, 1, 2, 5, and 10 years were included for analysis. Extracted implant characteristics included implant design, coating, and surgical approach. Data was analyzed using a random effects model.

**Results:**

73 studies on 120 cohorts and 2,970 uncemented stems were included. 119 cohorts reported on subsidence and 91 on retroversion. The pooled subsidence at 3 months was 0.29 mm (95% confidence interval [CI] 0.19–0.39) and 0.32 mm (CI 0.21–0.43) at 2 years. The pooled retroversion at 3 months was 0.51° (CI 0.33–0.70) and 0.70° (CI 0.48–0.93) at 2 years. Hydroxyapatite-coated stems showed the least migration (subsidence 0.26 mm; CI 0.13–0.40; retroversion 0.51°; CI 0.22–0.80) among different coating types. The anterior approach showed more migration (subsidence 1.04 mm, CI 0.53–1.55; retroversion 1.52°, CI 1.08–1.95) than other surgical approaches.

**Conclusion:**

Our study shows that most subsidence and retroversion of uncemented femoral stems occurs during the first 3 months. Stabilization of subsidence occurred after 3 months, and retroversion stabilized after 2 years. Migration patterns differ based on stem type, coating, surgical approach, the time period when inclusion started, and timing of baseline measurement.

Each year, more than 1 million primary total hip replacements (THR) are performed worldwide, and this number is expected to continue to rise [1]. The overall survival of THRs has improved over the last decades, but aseptic loosening remains one of the main causes for late revision [2]. Aseptic loosening can be predicted by early migration patterns measured with radiostereometric analysis (RSA) [3]. Early migration patterns have been systematically reviewed for acetabular components in hip arthroplasty [4]. Van der Voort et al. (2015) examined the association between early migration and late revision of 14 prosthesis and fixation combinations and found an association between early subsidence and aseptic loosening for cemented shape-closed stems, but no clear association was found for uncemented stems [5]. For femoral stems, subsidence and retroversion are considered the most important types of migration because known failing stems have shown high subsidence and retroversion. Subsidence and retroversion are the directions of the loading of the stem, and are thought to predict aseptic loosening [5-8]. It is important to first determine the RSA migration patterns of femoral stems before they can be combined with survival data to establish thresholds. Therefore, in this review, we aim to answer the following research questions: What is the RSA migration pattern of uncemented hip stems in primary total hip replacement (THR) in terms of subsidence and retroversion? What is the RSA migration pattern of uncemented stems according to implant, patient, and study characteristics?

## Methods

This systematic review and meta-analysis is performed in accordance with the Preferred Reporting Items for Systematic Reviews and Meta-Analyses (PRISMA) statement and a protocol was registered online on March 12, 2024, prior to the start of the review (https://osf.io/bhpr4) [9]. During data extraction, we added some variables to the final protocol (https://osf.io/hze9m). No other changes were made and the outcomes and analyses described in the protocol are all reported in this paper.

### Literature search

Based on our experience with radiostereometric analysis (RSA) literature reviews [5,10-12], the search strategy was composed of a combination of the terms defining “RSA” and “Joint Replacement” (Appendix A, see Supplementary data). We searched articles up to February 22, 2024, in PubMed, Web of Science, Cochrane, and Embase.

### Study selection

The inclusion criteria were: (i) primary uncemented THR with explicitly stated prosthesis; (ii) subsidence and/or retroversion measured with RSA; and (iii) at least 2 postoperative follow-up RSA measurements within the first 2 years in addition to the postoperative baseline. Studies with fewer than 5 THRs per prothesis, non-clinical studies (e.g., phantom or animal studies, editorials, and reviews), studies with more than 5% fractures at baseline and studies not available in full text were excluded. If the same cohort was reported in multiple publications, the publication with the longest follow-up was formally included, while the other publications were used for additional data if required (e.g., earlier follow-up, baseline information).

All RSA studies that reported on migration after uncemented primary THR were identified. All steps of the review were performed by 1 reviewer and re-examined by a team of second reviewers. The screening of titles and abstracts and the full-text screening was performed in duplicate. The initial screening of the titles and abstracts was performed by LW, and the second screening was performed by a team of reviewers (BP and CR) using machine learning software (ASReview 1.5.1; https://asreview.nl/) to determine the order in which publications were screened [13]. The use of machine learning assisted reviewing has been validated in orthopedics [14]. In the case of disagreement, the study remained eligible for the full-text screening. The full text of the eligible studies was then reviewed by LW and the team of second reviewers (BP and CR). Any disagreements were discussed and resolved.

### Data extraction

The data from the included studies was extracted by LW. A team of second reviewers (BK, BP, CR, and TG) checked the data extraction for any inconsistencies in a subset of 10 randomly selected articles. An SPSS database (IBM SPSS Statistics 26.0; IBM Corp, Armonk, NY, USA) was constructed using 5 randomly selected studies that were included in the review. The implant design was classified according to Radaelli et al. [15], who divided the uncemented stems into 6 design types (flat taper, quadrangular taper, fit-and-fill, conical, cylindrical, and calcar-guided ultra-short neck-preserving stems). Stems that did not fit in any of the 6 groups were classified as “Other.” The stems were also categorized based on coating. A stem was classified as hydroxyapatite (HA) coated if any part of its surface area was HA-coated. If part of the stem was coated with a porous coating (not HA), it was classified as porous coated. Stems with neither coating were classified as uncoated. The non-weightbearing group had no weightbearing before the baseline measurement; other studies were included in the weightbearing group, even with partial weightbearing. Only precision measurements calculated from double examinations within the sample of the study were included. Data was extracted from the manuscript text, tables, and figures (using WebPlotDigitizer version 4.8 [16,17]). The mean RSA migration, standard deviation (SD), and precision (SD) was extracted or calculated from reported median, interquartile range (IQR), confidence interval (CI), range, or standard error (SE) using internationally accepted methodology [18-28]. Subsidence and retroversion are reported as positive values in this review. In the rare case that only the mean migration was given without IQR, CI, range, SE, or SD, the SD was calculated as the average SD from similar studies [10,19,29]. For this, we used studies with the same prosthesis, or (if unavailable) studies with implants of similar design. Instead of excluding data, which could lead to bias, we performed sensitivity analyses to check the validity of the imputed data.

### Data synthesis and analysis

In line with previous reviews, we determined and plotted the 10th, 25th, 50th, 75th, and 90th percentile of the means up to 2 years. There were not enough study cohorts with follow-up beyond 2 years to reliably determine percentiles for additional follow-up moments. A random effects model (restricted maximum likelihood estimator) was used to pool the migration of individual study cohorts in order to estimate the overall migration for each follow-up and its associated 95% confidence interval [10,29]. We employed a random effects meta-regression on study level covariates such as coating type, stem type, and the start of inclusion of RSA studies [30]. Patient factors such as age and percentage of females were examined within the most frequently reported stem. All analyses were performed using Metafor Package R statistics (R Foundation for Statistical Computing, Vienna, Austria) [30]. The influence of publication bias was assessed with funnel plots and comparison of the results with the revision rates of the Dutch national joint registry (LROI). According to the RSA guidelines, the recommended baseline measurement takes place within 2 weeks after surgery, to avoid missing initial migration [31]. To examine the possible effect of studies with a delayed baseline measurement or an imputed SD, we compared the overall mean subsidence and retroversion at the 2-year follow-up with and without those studies included. If studies with a delayed baseline measurement or imputed SD significantly affected the migration results, these studies were excluded from the analyses. Studies with similar characteristics were examined to check for overlap of included patients. If studies had a clear overlap, only the publication with the largest cohort and longest follow-up was included. When potential overlap between publications could not be definitively excluded, we conducted a sensitivity analysis in which results obtained by including all potentially overlapping reports were compared with those obtained by including only the publication with the largest cohort and longest follow-up.

In the graphs, the uncoated ProxiLock migration pattern was plotted to show an example migration pattern of a failed uncemented stem, but should not be used as a threshold [8]. The ProxiLock was chosen as an example because it shows excessive migration resulting in a high percentage of revisions [8].

### Quality assessment

The quality of the included studies was appraised using the modified Assessment of Quality In Lower limb Arthroplasty (AQUILA) methodological score [32]. Similar to previous RSA reviews, we used only the items of the AQUILA score considered relevant for appraisal of (early) migration: cohort construction, predefinition of follow-up, and loss-to-follow-up. For the loss-to-follow-up we did not count study endpoints such as failures or deaths. Higher scores indicate higher quality with a maximum score of 7 [5,10-12].

### Data sharing plan, funding, use of AI, and disclosures

The database is available at https://osf.io/4w2ut/files/ze7g6.

This study is part of the PhD project of LW, which is funded by an external funder (Implant Preservation Devices). The external funder had no influence on this study.

During screening, machine learning software (ASReview 1.5.1; https://asreview.nl/) was used to determine the order in which publications were screened. ChatGPT was used for copy-editing author-written sentences, to substitute a limited number of words in individual author-selected sentences to improve sentence flow and reduce repetitive use of individual words. We used Microsoft Word (Microsoft, Redmond, WA) for manuscript preparation, which may have included AI in some of its proofing tools. No AI was used in the preparation of figure or tables, data analysis, or interpretation of the results.

Authors declare no conflict of interests. Complete disclosure of interest forms according to ICMJE are available on the article page, doi: 10.2340/17453674.2026.45412

## Results

### Inclusion of RSA studies

**Figure 1:**
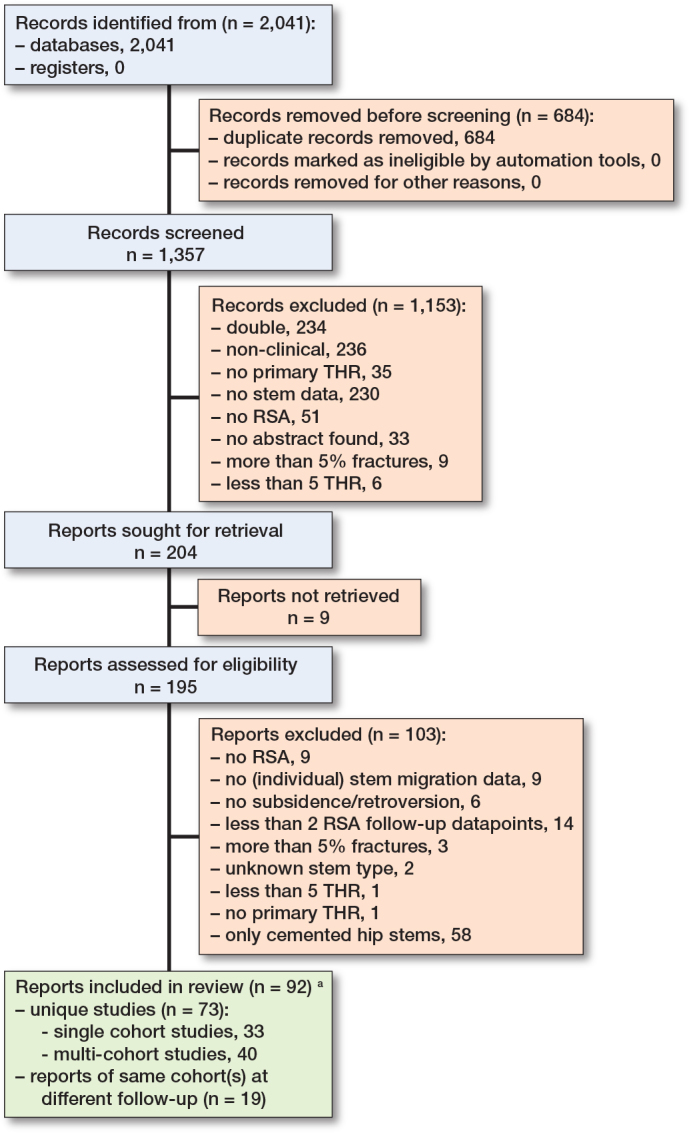
PRISMA flowchart with selection process of the review. THR: total hip replacement; RSA: radiostereometric analysis. ^**a**^ Some cohorts are followed over time with findings published in multiple reports; therefore, the number of reports exceeds the number of unique studies.

2,041 records were identified with the search. After full-text screening, 92 reports on 73 unique studies were included (Appendix B, see Supplementary data). The included studies were published between 1988 and 2024 (median 2017). 120 cohorts with 2,970 stems at baseline and 66 different uncemented hip stems were included (Figure 1). The included studies had a median mean patient age of 59 years, a median of 50% female participants and median of 98% primary osteoarthritis (OA) (Table 1). The most reported follow-up measurement was at 1 year with 117 cohorts on subsidence and 84 cohorts on retroversion (Table 2).

**Table 1:** Study characteristics of the included studies. The mean, confidence interval (CI), median, and range are calculated across the study-reported means (unweighted by sample size)

	Mean (CI)	Median	Range
Age (years)	59.5 (58.3–60.6)	59	37.0–70.6
Female (%)	51.6 (47.9–55.3)	50	7.7–100
Primary OA (%)	88.3 (82.6–93.9)	97.5	0–100
Precision			
subsidence (mm)	0.26 (0.17–0.34)	0.15	0.03–1.37
retroversion (degrees)	1.36 (0.97–1.76)	0.95	0.10–4.45
Quality score	4.6 (4.3–4.9)	5	2–7

**Table 2:** Number of hips and cohorts per follow-up measurement

	Follow-up, months							
	Baseline	1.5	3	6	12	24	60	120
Cohorts								
subsidence	119	39	92	82	117	105	36	4
retroversion	91	24	68	49	84	79	27	4
Hips, n	2,970	803	2,094	1,925	2,661	2,409	840	66

### Migration results

**Figure 2:**
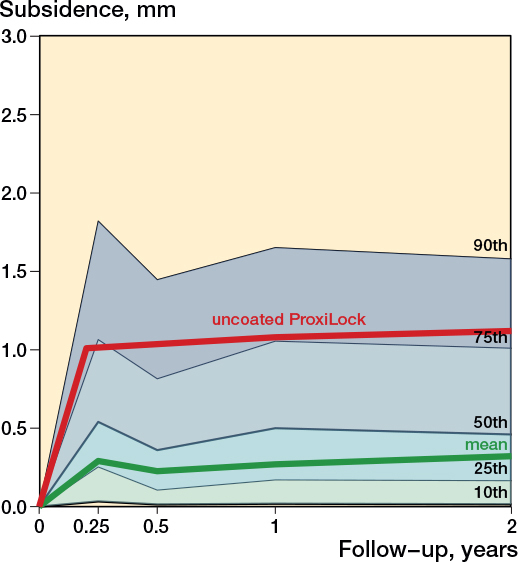
Mean pooled subsidence pattern of uncemented stems and percentiles among included cohorts, with the known failure uncoated ProxiLock for reference [8].

**Figure 3:**
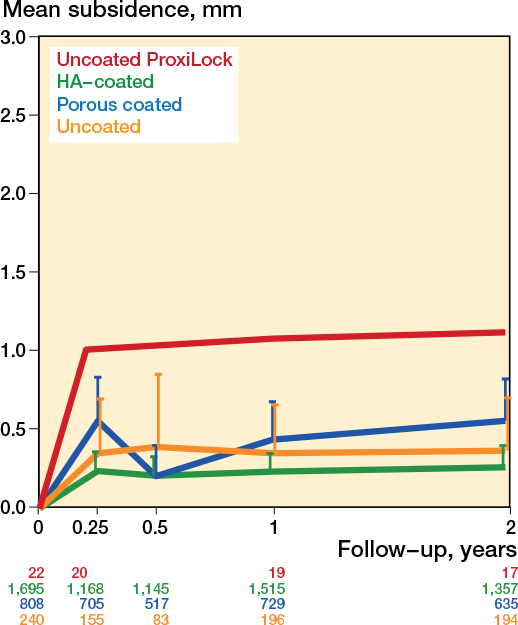
Mean pooled subsidence of HA-coated, porous-coated, and uncoated stems, with the known failure uncoated ProxiLock for reference [8]. The error bars represent the upper limit of the 95% confidence intervals, and the numbers below the graph indicate the number of hips per group at each follow-up.

Subsidence of the uncemented femoral stems occurred mostly within the first 3 months, after which the stems showed stabilization (Figure 2). The pooled subsidence at 3 months was 0.29 mm (CI 0.19–0.39; 92 cohorts) and 0.32 mm (CI 0.21–0.43; 99 cohorts) at 2 years. The subsidence of the uncemented stems between 3 months and 2 years was negligible (0.03 mm, CI –0.01 to 0.07; 80 cohorts). The subsidence pattern of the first 2 years showed differences between coatings; HA-coated stems showed the least subsidence (Figure 3 and Table C1, see Supplementary data). In the long term, femoral stems remained stable in terms of subsidence, with a pooled mean subsidence between 2 and 5 years of 0.03 mm (CI 0.00–0.07; 35 cohorts). Only 3 studies had a 10-year follow-up (Figure C1, see Supplementary data).

**Figure 4:**
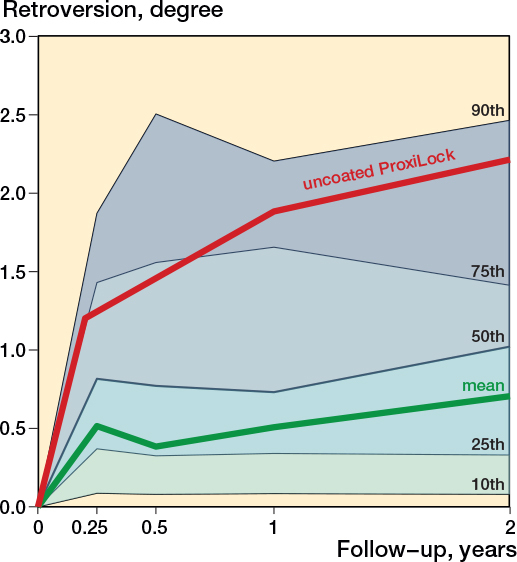
Mean pooled retroversion pattern of uncemented stems and percentiles among included cohorts, with the known failure uncoated ProxiLock for reference [8].

**Figure 5:**
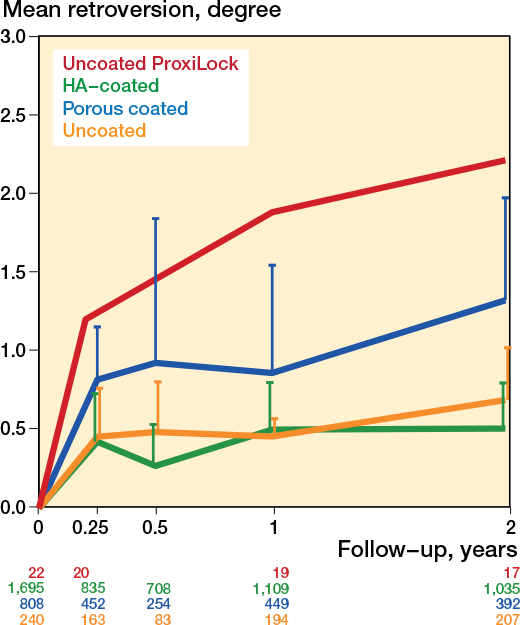
Mean pooled retroversion of HA-coated, porous-coated and uncoated stems, with the known failure uncoated ProxiLock for reference [8]. For error bars and numbers below the graph, see Figure 3.

Retroversion mostly occurred within the first 3 months and showed a minimal increase until 2 years (Figure 4). The mean pooled 3-month retroversion was 0.51° (CI 0.33–0.70; 68 cohorts) and the mean pooled 2-year retroversion was 0.70° (CI 0.48–0.93; 75 cohorts). Retroversion increased with a mean pooled 0.20° (CI 0.07–0.33; 56 cohorts) between 3 months and 2 years. Retroversion within the first 2 years revealed differences between coatings; HA-coated stems had the least retroversion (Figure 5 and Table C1, see Supplementary data). Long-term results showed a negligible retroversion of the stems over time, with a pooled mean retroversion between 2 and 5 years of –0.05° (CI –0.21 to 0.10; 27 cohorts). Only 3 studies measured retroversion at 10 years (Figure C2, see Supplementary data).

### Subgroups

**Figure 6:**
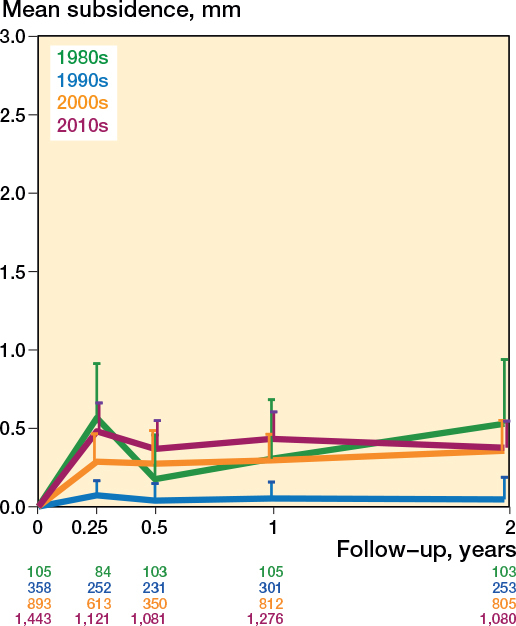
Pooled mean subsidence during the first 2 years based on the start of the inclusion period. For error bars and numbers below the graph, see Figure 3.

**Figure 7:**
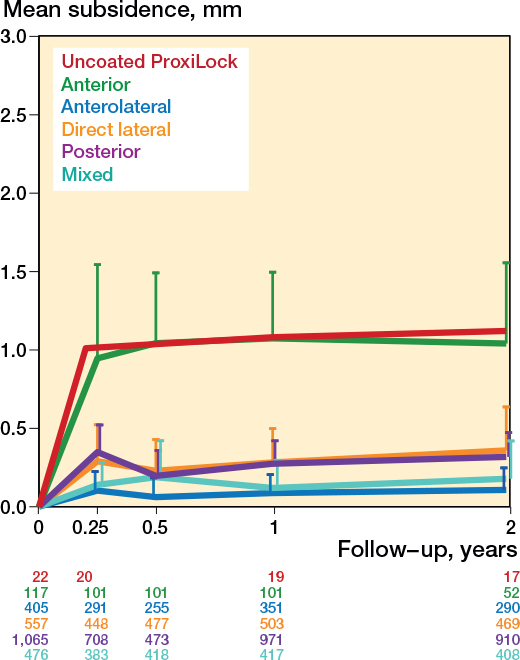
Pooled mean subsidence during the first 2 years per surgical approach, with the known failure uncoated ProxiLock for reference [8]. For error bars and numbers below the graph, see Figure 3.

Different stem types show different subsidence patterns, but the flat-taper, quadrangular taper, and fit-and-fill stems showed a similar pattern (Table C1 and Figure C3, see Supplementary data). Figure 6 shows that subsidence has increased over the last decades; the studies that started inclusion in the 1990s showed the least migration, after which it increased with each following decade (Table C1, see Supplementary data). Differences in subsidence patterns were observed between surgical approaches (Figure 7), with stems inserted through the anterior approach subsiding most (Table C1, see Supplementary data). There were no obvious differences in subsidence patterns based on RSA technique, randomization, and weightbearing (Figures C4–6, see Supplementary data). The mean age and the percentage of females were no effect modifiers on the subsidence patterns of the most frequently reported stem (Taperloc).

**Figure 8:**
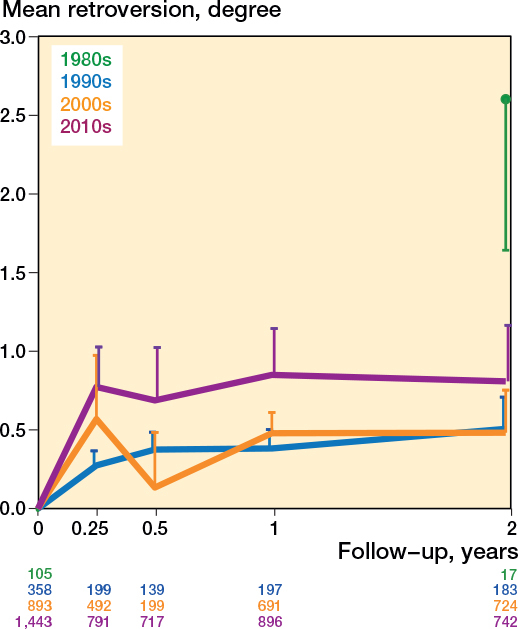
Pooled mean retroversion during the first 2 years based on the start of the inclusion period. For the 1980s studies there was no retroversion pattern available, so the 2-year retroversion result only is presented with the error bar showing the lower limit of the 95% confidence interval.

**Figure 9:**
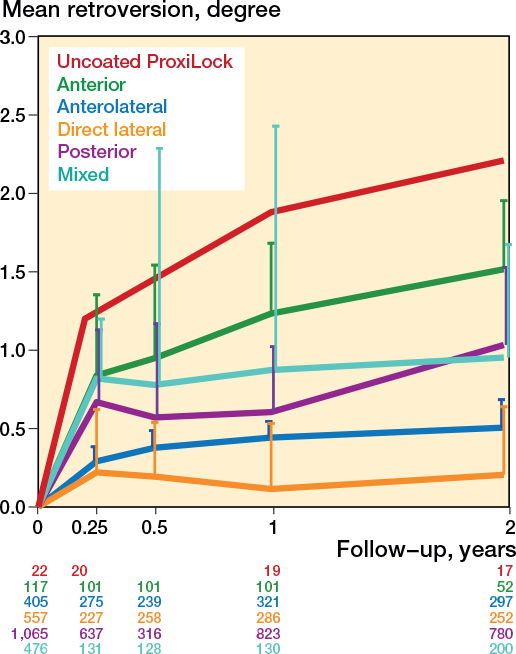
Pooled mean retroversion during the first 2 years per surgical approach, with the known failure uncoated ProxiLock for reference [8]. For error bars and numbers below the graph, see Figure 3.

The retroversion pattern of all the stem types showed considerable variation (Figure C7, see Supplementary data); however, the retroversion patterns of the flat-taper, quadrangular taper, and fit-and-fill stems were similar (Table C1, see Supplementary data). Figure 8 presents the retroversion patterns of the inclusion period per decade; the pooled mean retroversion was highest in the 1980s and lowest in the 1990s and 2000s (Table C1, see Supplementary data). The retroversion patterns of different surgical approaches are presented in Figure 9, where the anterior approach shows the most retroversion (Table C1, see Supplementary data). The retroversion patterns based on RSA technique, randomization, and weightbearing showed no obvious differences (Figures C8–10, see Supplementary data). The mean age and the percentage of females were no effect modifiers on the retroversion patterns of the most frequently reported stem (Taperloc).

### Sensitivity analyses and publication bias

**Figure 10:**
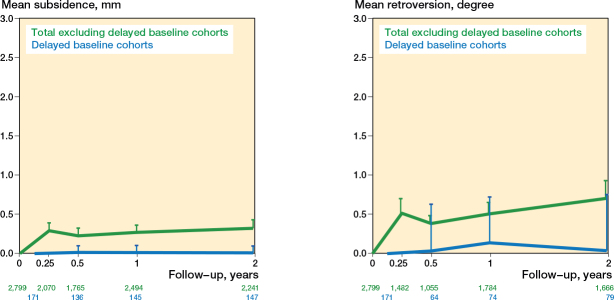
Effect of the delayed baseline studies on the pooled mean subsidence (left) and retroversion (right) during the first 2 years. The results of the cohorts with a delayed baseline start at 6 weeks on the graph. For error bars and numbers below the graph, see Figure 3.

The imputed SD and the studies with possible overlap had no significant effect on the migration results, but the studies with a delayed baseline measurement had a significantly lower overall mean subsidence and retroversion (Figure 10), so these 5 studies (6 cohorts, 171 hips) were not included in the migration analyses. Including the cohorts with a delayed baseline would have significantly lowered our results (Figure 10; Table C1, see Supplementary data). To assess publication bias, we compared our findings with the revision rates of the Dutch national joint registry (LROI) and used a funnel plot of subsidence and retroversion at the 1-year follow-up (most reported follow-up). The revision rates of the LROI were in line with our migration results; the most-commonly used stem types (flat-taper, quadrangular taper, and fit-and-fill stems) had similar migration patterns and also similar revision percentages [33]. The funnel plots were asymmetrical and had fewer datapoints on the left side of the funnel plot (Figures C11 and C12, see Supplementary data). In RSA, negative results are rarely seen for subsidence and retroversion, which should be considered when interpreting these funnel plots. More specifically, mean lower migration values tended to have smaller SDs, which results in greater weight in the funnel plot. This causes the center line to shift left and an uneven distribution of the data points. For this reason, the observed asymmetry was considered not typical of publication bias and most likely the result of the migration direction.

### Reported precision

Precision was reported as SD of double examinations. 50 of all 73 studies (68%) reported precision data on subsidence, but only 41 of these studies measured retroversion and reported its precision. The remaining 23 studies did not report precision measurements based on double examinations. The median precision of subsidence was 0.16 mm, and 1.06° for retroversion (see Table 1).

### Methodological quality

The included studies had a median quality score of 5 out of 7. All included studies followed a predefined follow-up schedule. The construction of the cohorts, however, was consecutive in 19 studies, non-consecutive in 9 studies, and unknown in the remaining studies (n = 45). The follow-up was fully completed in 27 studies, 20 studies had a maximum of 5% loss-to-follow-up, 22 studies had more than 5% loss-to-follow-up, and 4 studies did not mention the number of dropouts.

## Discussion

In this review, we aggregated subsidence and retroversion data of 2,970 uncemented hip stems and found that most migration occurs within the first 3 months. Afterwards, subsidence of the stems stabilized, whereas retroversion continued until stabilization at 2 years. Our results suggest that most subsidence and retroversion can be determined at 3 months. The stabilization of subsidence can be determined at a 1- or 2-year follow-up, while the stabilization of retroversion might need an additional later follow-up moment for assessment. The stems remained stable up to 5 years.

An interesting finding in this systematic review is that the femoral stems showed an initial overshoot of subsidence within the first 6 months. The initial subsidence is not surprising, but it is surprising that the initial subsidence seems to be followed by a slight proximal migration. This proximal migration could be caused by incorrect measurements, movement of the markers, or the results of a rotation or migration along another axis, but these are unlikely on a large scale. Another (hypothetical) cause is an overshoot reaction of the bone occurring between 3 and 6 months postoperatively.

### Subgroups

We identified different migration patterns for different stem types, but the most commonly used stem types (flat-taper, quadrangular taper, and fit-and-fill stems) showed similar migration patterns. The coating was the most important effect modifier, while mean age, percentage of females, and surgical approach were not associated with the migration patterns of fit-and-fill stems and Bi-Metric stems. More studies are needed to better understand the migration patterns of less-common stem types (cylindrical, conical, ultra-short neck preserving, and the “other” stems). The different surgical approaches also showed different migration patterns, where the anterior approach migrated the most. The longer learning curve associated with the anterior approach is unlikely to explain this finding, as this approach was performed exclusively by experienced surgeons, and prior studies have reported an increased risk of aseptic loosening for this approach [34,35]. Within the different stem types and within the most frequently reported stem (Taperloc), the cohorts with an anterior surgical approach showed more migration than the other cohorts. The studies that started in the 1980s show the most migration by far, but the migration seems to increase over the last decades. There is likely heterogeneity between the different decades regarding stem type and coating, which show different migration patterns across their respective subgroups. The migration patterns of RSA technique, randomization, and weightbearing showed no obvious differences, which is in line with previous literature [4,29,36]. The age and the percentage of females were not effect modifiers on the migration patterns of the most frequently reported stem (Taperloc).

### Sensitivity analyses and publication bias

We imputed SDs for 6 cohorts (3 studies/108 stems) due to missing SD data, which had no significant effect on our results. Nevertheless, it is important to present complete results as is also recommended in the updated RSA guidelines [31].

A delayed baseline missed the majority of early migration and therefore showed minimal migration, supporting the updated RSA guidelines’ recommendation for a baseline measurement within 2 weeks [31]. We excluded these cohorts prior to performing the migration analyses to prevent bias. A delayed baseline can still be used to assess long-term stability, but the overall migration pattern cannot be compared due to the missed early migration.

### Precision and methodological quality

Many articles had incomplete information, especially regarding follow-up and precision. Reasons for loss to follow-up were often missing, and sometimes the follow-up and loss-to-follow-up numbers were not reported at all. Precision was often missing, reported without specifying the metric (SD, 95% CI, etc.), or not measured according to the RSA guidelines (double examinations performed within the study population) [31]. To improve the quality and transparency of the studies, precision should be accurately measured and loss-to-follow-up should be clearly reported.

### Strengths

To reduce the likelihood of human error and enhance the reliability of the data and results, each step of this review and meta-analysis was completed or verified by at least 2 individuals. Data extraction could not be completed in duplicate due to the substantial workload involved in this part of the review but was inspected by a team of second reviewers instead. Additionally, not including the studies with a delayed baseline in the migration analyses increased the accuracy of our findings.

### Limitations of included studies

Overall, the majority of studies reported complete data, although some studies missed details (e.g., SDs, start of inclusion, loss-to-follow-up). Another limitation of the included studies is the substantial heterogeneity in stem type, coating, surgical approach, and start of inclusion—factors whose respective subgroups have shown different migration patterns. Furthermore, only a few studies reported migration beyond 2 years and there is potential attrition bias if participants with high migration dropped out or were revised before the study endpoint.

### Limitations of this review

Due to missing data, we had to impute the missing SDs, start of inclusion, and number of hips at follow-up. Since the missing SDs occurred in only a small number of studies, its impact on our results is likely minimal. The imputation of the start of inclusion was necessary to examine migration trends over time. We minimized the effect of misclassification by grouping by decades. By imputing the number of hips per follow-up, we likely overestimated the true count, but probably not substantially.

14 studies did not report surgical indications, so the number of THRs for femoral fractures is unknown. Patients receiving a THR for a femoral fracture have different long-term results than osteoarthritis patients, especially regarding revision risk [37]. Therefore, we excluded studies with more than 5% fracture patients, to avoid bias in our results. Because it is unlikely that these 14 studies included many fracture patients without reporting it, the impact on our results is probably minimal.

We included studies that only reported their results in graphs. Data extracted from graphs is not as accurate as data from tables or text. However, by using a validated method, we can assume that the errors are minimal and did not affect our results [16].

Lastly, studies with an unknown timing of the baseline measurement were included for the migration analyses. Therefore, it cannot be entirely ruled out that some studies may have had a delayed baseline measurement (beyond 2 weeks postoperatively). This is unlikely, as it does not align with RSA practices of the involved research teams, which we know due to their other publications and international presentations on RSA studies.

### Conclusion

Most subsidence and retroversion occur within the first 3 months, with subsidence stabilizing thereafter and retroversion showing minimal further change up to 2 years. Our study shows that migration (subsidence and retroversion) patterns differ based on stem type, coating, surgical approach, start of inclusion, and whether the baseline measurement was performed within 2 weeks postoperatively or not. These migration patterns could be used as a reference for future studies and may be used for the development of thresholds. The next step is combining these results with survival data to establish migration thresholds, which can be used to evaluate the safety of new implants introduced to the market.

### Supplementary data

Search strategy, list of included studies, Table C1, and Figures C1–C16 are available as Supplementary data on the article page, doi: 10.2340/17453674.2026.45412

## Supplementary Material



## Notes

BP and RN conceived and designed the study. All authors contributed to finalizing the methodology. LW collected the data. CR, TG, BK, and BP validated the data. LW performed statistical analyses. CR, TG, BK, and BP provided critical revisions to the manuscript. All authors contributed to drafting and revising the manuscript and approved the final version for submission.

Handling co-editors: Keijo Mäkelä and Robin Christensen

*Acta* thanks Hannu T Aro and Johan Kärrholm for help with peer review of this manuscript.
