# Supplementary material for: Migration patterns of uncemented femoral stems in hip replacement: a systematic review and meta-analysis of clinical radiostereometric analysis cohort studies
Source: Acta Orthop. 2026 Apr 16;97:247–54. doi: 10.2340/17453674.2026.45412 (PMC13085547; doi:10.2340/17453674.2026.45412)
Supplement: Supplementary file 1 [file ActaO-97-45412-s1.pdf]

SUPPLEMENTARY DATA

Appendix A: search strategy

PubMed

((“Photogrammetry”[Mesh:NoExp] OR Radiostereometric Analysis [Mesh] OR “roentgen stereophotogrammetric analysis”[tiab] OR “RSA” [tiab] OR “Radiostereometric” [tiab] OR “Radiostereometrics” [tiab] OR “stereophotogrammetric” [tiab] OR “stereophotogrammetrics” [tiab] OR “stereophotogram-metry” [tiab] OR “stereo-photogrammetric” [tiab] OR “stereophotogrammetrics” [tiab] OR “stereophotogrammetry” [tiab] OR “Photofluorography” [Mesh] OR “roentgen fluoroscopic”[tiab] OR “roentgen fluoroscopics”[tiab] OR “roentgen fluoroscopy”[tiab]))

AND

((“Joint Prosthesis”[Mesh:NoExp] OR “Hip Prosthesis”[Mesh] OR “hip prosthesis”[tiab] OR “hip prostheses”[tiab] OR “prosthetic hip”[tiab] OR “THA”[tiab] OR “THR”[tiab] OR “TKR”[tiab] OR “joint replacement”[tiab] OR “Arthroplasty, Replacement”[mesh:NoExp] OR “total hip replacement”[tiab] OR “total hip arthroplasty”[tiab] OR “Arthroplasty, Replacement, Hip”[Mesh]))

February 22, 2024 => 731 hits

Web of Science

TS=(“Photogrammetr*” OR “RSA” OR “radiostereometr*” OR “radio-stereometr*” OR “stereophotogram-metr*” OR “stereo-photogrammetr*” OR “roentgen fluoroscop*” OR “Photofluorograph*” OR “Photo-flu-orograph*”)

AND

TS=(“Joint Prosthe*” OR “hip prosthe*” OR THA OR THR OR “hip arthroplast*” OR “hip replacement*”)

February 22, 2024 => 613 hits

Of which 401 unique imported

Cochrane database

(“Photogrammetr*” OR “RSA” OR “radiostereometr*” OR “radio-stereometr*” OR “stereophotogrammetr*” OR “stereo-photogrammetr*” OR “roentgen fluoroscop*” OR “Photofluorograph*” OR “Photo-fluorograph*”):ti,ab,kw

AND

(“Joint Prosthe*” OR “hip prosthe*” OR THA OR THR OR “hip arthroplast*” OR “hip replacement*”):ti,ab,kw

February 22, 2024 => 116 hits

Of which 65 unique imported

Embase

(Stereophotogrammetry/ OR exp radiostereometric analysis/ OR “RSA”.ti,ab. OR “Radiostereometr*”.ti,ab. OR “Radio-stereometr*”.ti,ab. OR “stereophotogrammetr*”.ti,ab. OR “stereo-photogrammetr*”.ti,ab. OR exp fluorography/ OR “Photofluorograph*”.ti,ab. OR “Photo-fluorograph*”.ti,ab. OR “roentgen fluoroscop*”.ti,ab.)

AND

(joint prosthesis/ OR exp hip prosthesis/ OR “hip prosthe*”.ti,ab. OR “hip arthroplast*”.ti,ab. OR “hip replacement*”.ti,ab. OR “prosthetic hip*”.ti,ab. OR “THA”.ti,ab. OR “THR”.ti,ab. OR “joint replacement*”.ti,ab. OR replacement arthroplasty/ OR exp hip replacement/) NOT (conference OR conference abstract OR “conference review”).pt.

February 22, 2024 => 581 hits

Of which 314 unique imported

Total hits without de-doubling February 22, 2024 = 731 + 613 + 116 + 581 = 2041

Total hits with de-doubling February 22, 2024 = 731 + 401 + 65 + 314 = 1511

After de-doubling within Endnote another 154 were removed => 1357

Appendix B: Included studies

1. Acklin YP, Jenni R, Bereiter H, Thalmann C, Stoffel K. Prospective clinical and radiostereometric analysis of the Fitmore short-stem total hip arthroplasty. Arch Orthop Trauma Surg. 2016; 136(2): 277-84. doi: 10.1007/s00402-015-2401-9

2. Alsousou J, Oragu E, Martin A, Strickland L, Newman S, Kendrick B, et al. Primary stability of a proximally coated and tapered stem a two-year radiostereometric analysis. Bone Joint J. 2021; 103B(4): 644-9. doi: 10.1302/0301-620X.103B4.BJJ-2020-1648.R1

3. Aro E, Alm JJ, Moritz N, Mattila K, Aro HT. Good stability of a cementless, anatomically designed femoral stem in aging women: a 9-year RSA study of 32 patients. Acta Orthop. 2018; 89(5): 490-5. doi: 10.1080/17453674.2018.1490985

4. Aro HT, Nazari-Farsani S, Vuopio M, Löyttyniemi E, Mattila K. Effect of denosumab on femoral periprosthetic BMD and early femoral stem subsidence in postmenopausal women undergoing cementless total hip arthroplasty. JBMR Plus. 2019; 3(10): e10217. doi: 10.1002/jbm4.10217

5. Baad-Hansen T, Kold S, Olsen N, Christensen F, Søballe K. Excessive distal migration of fiber-mesh coated femoral stems. Acta Orthop. 2011; 82(3): 308-14. doi: 10.3109/17453674.2011.574562

6. Boe BG, Rohrl SM, Heier T, Snorrason F, Nordsletten L. A prospective randomized study comparing electrochemically deposited hydroxyapatite and plasma-sprayed hydroxyapatite on titanium stems: 55 hips followed for 2 years with RSA and DXA. Acta Orthop. 2011; 82(1): 13-9. doi: 10.3109/17453674.2010.548027

7. Bottner F, Zawadsky M, Su EP, Bostrom M, Palm L, Ryd L, et al. Implant migration after early weightbearing in cementless hip replacement. Clin Orthop Relat Res. 2005; 436: 132-7. doi: 10.1097/01.blo.0000160380.15429.fb

8. Budde S, Seehaus F, Schwarze M, Hurschler C, Floerkemeier T, Windhagen H, et al. Analysis of migration of the Nanos® short-stem hip implant within two years after surgery. Int Orthop. 2016; 40(8): 1607-14. doi: 10.1007/s00264-015-2999-9

9. Carlsson LV, Albrektsson BEJ, Albrektsson BG, Albrektsson TO, Jacobsson CM, Macdonald W, et al. Stepwise introduction of a bone-conserving osseointegrated hip arthroplasty using RSA and a randomized study: I. Preliminary investigations - 52 patients followed for 3 years. Acta Orthop. 2006; 77(4): 549-58. doi: 10.1080/17453670610012601

10. Carlsson LV, Albrektsson T, Albrektsson BEJ, Jacobsson CM, Macdonald W, Regner L, et al. Stepwise introduction of a bone-conserving osseointegrated hip arthroplasty using RSA and a randomized study: II. Clinical proof of concept - 40 patients followed for 2 years. Acta Orthop. 2006; 77(4): 559-66. doi: 10.1080/17453670610012610

11. Christensson A, Nemati HM, Flivik G. Comparison between model-based RSA and an AI-based CT-RSA: An accuracy study of 30 patients. Acta Orthop. 2024; 95: 39-46. doi: 10.2340/17453674.2024.35749

12. Christiansen JD, Ejaz A, Nielsen PT, Laursen M. An ultra-short femoral neck-preserving hip prosthesis: A 2-year follow-up study with radiostereometric analysis and dual x-ray absorptiometry in a stepwise introduction. J Bone Joint Surg Am. 2020; 102(2): 128-36. doi: 10.2106/jbjs.19.00104

13. Ciccotti MG, Rothman RH, Hozack WJ, Moriarty L. Clinical and roentgenographic evaluation of hydroxyapatite-augmented and nonaugmented porous total hip arthroplasty. J Arthroplasty. 1994; 9(6): 631-9. doi: 10.1016/0883-5403(94)90117-1

14. Coffey SP, Sorial RM, Sharma R, Field JR. Two-year migration characteristics of a novel cementless femoral stem: A radiostereometric analysis and clinical outcomes study. ANZ J Surg. 2021; 91(3): 398-403. doi: 10.1111/ans.16616

15. Collopy D, Manara J. Two-year prospective RSA measured migration of a third-generation taper wedge cementless femoral stem. Surg Technol Int. 2022; 41: 321-8. doi: 10.52198/22.Sti.41.Os1572

16. Critchley O, Callary S, Mercer G, Campbell D, Wilson C. Long-term migration characteristics of the Corail hydroxyapatite-coated femoral stem: A 14-year radiostereometric analysis follow-up study. Arch Orthop Trauma Surg. 2020; 140(1): 121-7. doi: 10.1007/s00402-019-03291-8

17. de Waard S, Sierevelt IN, Jonker R, Hoornenborg D, van der Vis HM, Kerkhoffs GMMJ, et al. The migration pattern and initial stability of the Optimys short stem in total hip arthroplasty: A prospective 2-year follow-up study of 33 patients with RSA. Hip Int. 2021; 31(4): 507-15. doi: 10.1177/1120700020901844

18. Dyreborg K, Andersen MR, Winther N, Solgaard S, Flivik G, Petersen MM. Migration of the uncemented Echo Bi-Metric and Bi-Metric THA stems: A randomized controlled RSA study involving 62 patients with 24-month follow-up. Acta Orthop. 2020; 91(6): 693-8. doi: 10.1080/17453674.2020.1802682

19. Edmondson M, Ebert J, Nivbrant O, Wood D. Prospective randomised clinical trial assessing the subsidence and rotation of two modular cementless femoral stems (Global K2 and Apex). J Orthop. 2014; 11(2): 96-102. doi: 10.1016/j.jor.2014.02.001

20. Ferguson RJ, Broomfield JA, Malak TT, Palmer AJR, Whitwell D, Kendrick B, et al. Primary stability of a short bone-conserving femoral stem: A two-year randomized controlled trial using radiostereometric analysis. Bone Joint J. 2018; 100-b(9): 1148-56. doi: 10.1302/0301-620x.100b9.Bjj-2017-1403.R1

21. Floerkemeier T, Budde S, Lewinski GV, Windhagen H, HurSchler C, Schwarze M. Greater early migration of a short-stem total hip arthroplasty is not associated with an increased risk of osseointegration failure: 5th-year results from a prospective RSA study with 39 patients, a follow-up study. Acta Orthop. 2020; 91(3): 266-71. doi: 10.1080/17453674.2020.1732749

22. Fontalis A, Kayani B, Vanhegan I, Tahmassebi J, Haddad IC, Giebaly DE, et al. 2-year radiostereometric analysis evaluation of a short, proximally coated, triple-taper blade femoral stem versus a quadrangular-taper stem with reinforced proximal body: A randomized controlled trial. J Arthroplasty. 2023; 38(7s): S152-s61. doi: 10.1016/j.arth.2023.03.030

23. Glassman AH, Crowninshield RD, Schenck R, Herberts P. A low stiffness composite biologically fixed prosthesis. Clin Orthop Relat Res. 2001; 393: 128-36. doi: 10.1097/00003086-200112000-00015

24. Grant P, Aamodt A, Falch JA, Nordsletten L. Differences in stability and bone remodeling between a customized uncemented hydroxyapatite coated and a standard cemented femoral stem A randomized study with use of radiostereometry and bone densitometry. J Orthop Res. 2005; 23(6): 1280-5. doi: 10.1016/j.orthres.2005.03.016.1100230607

25. Haugan K, Foss OA, Husby OS, Husby VS, Svenningsen S, Winther SB. Surgical approach had minor association with femoral stem migration in total hip arthroplasty: Radiostereometric analysis of 61 patients after 5-year follow-up. Acta Orthop. 2023; 94: 410-5. doi: 10.2340/17453674.2023.18264

26. Hjorth MH, Kold S, Søballe K, Langdahl BL, Nielsen PT, Christensen PH, et al. Preparation of the femoral bone cavity for cementless stems: Broaching vs compaction. A five-year randomized radiostereometric analysis and dual energy x-ray absorption study. J Arthroplasty. 2017; 32(6): 1894-901. doi: 10.1016/j.arth.2016.12.029

27. Hoornenborg D, Schweden AMC, Sierevelt IN, van der Vis HM, Kerkhoffs GMMJ, Haverkamp D. The influence of hydroxyapatite coating on continuous migration of a Zweymuller-type hip stem: A double-blinded randomised RSA trial with 5-year follow-up. Hip Int. 2023; 33(1): 73-80. doi: 10.1177/11207000211006782

28. Itayem R, Arndt A, Daniel J, McMinn DJW, Lundberg A. A two-year radiostereometric follow-up of the first generation Birmingham Mid Head Resection arthroplasty. Hip Int. 2014; 24(4): 355-62. doi: 10.5301/hipint.5000136

29. Kärrholm J, Anderberg C, Snorrason F, Thanner J, Langeland N, Malchau H, et al. Evaluation of a femoral stem with reduced stiffness. A randomized study with use of radiostereometry and bone densitometry. J Bone Joint Surg Am. 2002; 84(9): 1651-8. doi: 10.2106/00004623-200209000-00020

30. Karrholm J, Frech W, Nivbrant B, Malchau H, Snorrason F, Herberts P. Fixation and metal release from the Tifit femoral stem prosthesis: 5-Year follow-up of 64 cases. Acta Orthop Scand. 1998; 69(4): 369-78. doi: 10.3109/17453679808999049

31. Kärrholm J, Snorrason F. Subsidence, tip, and hump micromovements of noncoated ribbed femoral prostheses. Clin Orthop Relat Res. 1993; 287: 50-60.

32. Kent M, Edmondson M, Ebert J, Nivbrant N, Kop A, Wood D, et al. Stem migration and fretting corrosion of the antirotation pin in the K2/Apex hip system. J Arthroplasty. 2016; 31(3): 727-34. doi: 10.1016/j.arth.2015.10.004

33. Kiernan S, Geijer M, Sundberg M, Flivik G. Effect of symmetrical restoration for the migration of uncemented total hip arthroplasty: A randomized RSA study with 75 patients and 5-year follow-up. J Orthop Surg Res. 2020; 15(1): 225. doi: 10.1186/s13018-020-01736-0

34. Knudsen MB, Thillemann JK, Jørgensen PB, Jakobsen SS, Daugaard H, Søballe K, et al. Electrochemically applied hydroxyapatite on the cementless porous surface of Bi-Metric stems reduces early migration and has a lasting effect : An efficacy trial of a randomized five-year follow-up radiostereometric study. Bone Joint J. 2022; 104-b(6): 647-56. doi: 10.1302/0301-620x.104b6.Bjj-2021-1545.R1

35. Kok RY, Koster LA, Kaptein BL, Fiocco M, Keizer SB. A model-based radiostereometric analysis (RSA) randomized control trial evaluating the stability of the cementless Taperloc hip stem: The TapHip study 2-year follow-up RSA and PROMs results. Acta Orthop. 2022; 93: 212-21. doi: 10.2340/17453674.2021.1127

36. Kruijntjens DSMG, Koster L, Kaptein BL, Jutten LMC, Arts JJ, Ten Broeke RHM. Early stabilization of the uncemented Symax hip stem in a 2-year RSA study. Acta Orthop. 2020; 91(2): 159-64. doi: 10.1080/17453674.2019.1709956

37. Lindalen E, Dahl J, Nordsletten L, Snorrason F, Høvik Ø, Röhrl S. Reverse hybrid and cemented hip replacement compared using radiostereometry and dual-energy X-ray absorptiometry: 43 hips followed for 2 years in a prospective trial. Acta Orthop. 2012; 83(6): 592-8. doi: 10.3109/17453674.2012.742393

38. Luites JWH, Spruit M, van Hellemondt GG, Horstmann WG, Valstar ER. Failure of the uncoated titanium ProxiLock femoral hip prosthesis. Clin Orthop Relat Res. 2006; 448: 79-86. doi: 10.1097/01.blo.0000224011.12175.83

39. Mahmoud AN, Kesteris U, Flivik G. Stable migration pattern of an ultra-short anatomical uncemented hip stem: A prospective study with 2 years radiostereometric analysis follow-up. Hip Int. 2017; 27(3): 259-66. doi: 10.5301/hipint.5000458

40. Matejcic A, Vidovic D, Nebergall A, Greene M, Bresina S, Tepic S, et al. New cementless fixation in hip arthroplasty: A radiostereometric analysis. Hip Int. 2015; 25(5): 477-83. doi: 10.5301/hipint.5000254

41. McCalden RW, Korczak A, Somerville L, Yuan X, Naudie DD. A randomised trial comparing a short and a standard-length metaphyseal engaging cementless femoral stem using radiostereometric analysis. Bone Joint J. 2015; 97-b(5): 595-602. doi: 10.1302/0301-620x.97b5.34994

42. Munir S, Suzuki L, Dixon M. Migration characteristics of a proximally coated collarless femoral stem: A prospective 2-year radiostereometric analysis study. Arthroplast Today. 2023; 22: 101157. doi: 10.1016/j.artd.2023.101157

43. Nebergall AK, Rolfson O, Rubash HE, Malchau H, Troelsen A, Greene ME. Stable fixation of a cementless, proximally coated, double wedged, double tapered femoral stem in total hip arthroplasty: A 5-year radiostereometric analysis. J Arthroplasty. 2016; 31(6): 1267-74. doi: 10.1016/j.arth.2015.11.036

44. Nieuwenhuijse MJ, Valstar ER, Nelissen RGHH. 5-year clinical and radiostereometric analysis (RSA) follow-up of 39 CUT femoral neck total hip prostheses in young osteoarthritis patients. Acta Orthop. 2012; 83(4): 334-41. doi: 10.3109/17453674.2012.702392

45. Nieuwenhuijse MJ, Vehmeijer SBW, Mathijsen NMC, Keizer SB. Fixation of the short global tissue- sparing hip stem Two-year follow-up results of a randomized controlled clinical and roentgen stereophotogrammetric analysis study. Bone Joint J. 2020; 102B(6): 699-708. doi: 10.1302/0301-620X.102B6.BJJ-2019-1026.R2

46. Nistor L, Blaha JD, Kjellström U, Selvik G. In vivo measurements of relative motion between an uncemented femoral total hip component and the femur by roentgen stereophotogrammetric analysis. Clin Orthop Relat Res. 1991; 269: 220-7.

47. Nysted M, Foss OA, Klaksvik J, Benum P, Haugan K, Husby OS, et al. Small and similar amounts of micromotion in an anatomical stem and a customized cementless femoral stem in regular-shaped femurs. A 5-year follow-up randomized RSA study. Acta Orthop. 2014; 85(2): 152-8. doi: 10.3109/17453674.2014.899846

48. Nyström A, Kiritopoulos D, Mallmin H, Lazarinis S. Continuous periprosthetic bone loss but preserved stability for a collum femoris-preserving stem: Follow-up of a prospective cohort study of 21 patients with dual-energy X-ray absorptiometry and radiostereometric analysis with minimum 8 years of follow-up. Acta Orthop. 2022; 93: 206-11. doi: 10.2340/17453674.2021.1080

49. Okowinski M, Hjorth MH, Mosegaard SB, Jürgens-Lahnstein JH, Jakobsen SS, Christensen PH, et al. Ten-year comparison of two different techniques for femoral bone cavity preparation-broaching versus compaction in patients with cementless total hip arthroplasty A randomized radiostereometric study of 30 total hip arthroplasties in 15 patients operated bilaterally. Bone Jt Open. 2021; 2(12): 1035-42. doi: 10.1302/2633-1462.212.BJO-2021-0152.R1

50. Polus JS, Perelgut ME, Vasarhelyi EM, Teeter MG, Lanting BA. Femoral stem migration after direct lateral and direct anterior total hip arthroplasty: A prospective cohort study. Can J Surg. 2022; 65(4): E487-e95. doi: 10.1503/cjs.013221

51. Reiner T, Sonntag R, Kretzer JP, Clarius M, Jakubowitz E, Weiss S, et al. The migration pattern of a cementless hydroxyapatite-coated titanium stem under immediate full weight-bearing—A randomized controlled trial using model-based RSA. J Clin Med. 2020; 9(7): 1-10. doi: 10.3390/jcm9072077

52. Rilby K, Mohaddes M, Kärrholm J. Similar results after five years with the use of the Fitmore or the CLS femoral components. Bone Jt Open. 2023; 4(5): 306-14. doi: 10.1302/2633-1462.45.Bjo-2023-0007.R1

53. Rilby K, Mohaddes M, Nauclér E, Kärrholm J. Similar outcome with a new anteverted or a straight standard stem: A randomized study of 72 total hip arthroplasties evaluated with clinical variables, radiostereometry, and DXA up to 2 years. Acta Orthop. 2022; 93: 59-67. doi: 10.1080/17453674.2021.1993606

54. Rilby K, Nauclér E, Mohaddes M, Kärrholm J. No difference in outcome or migration but greater loss of bone mineral density with the Collum Femoris Preserving stem compared with the Corail stem: A randomized controlled trial with five-year follow-up. Bone Joint J. 2022; 104-b(5): 581-8. doi: 10.1302/0301-620x.104b5.Bjj-2021-1539.R1

55. Röhrl SM, Li MG, Pedersen E, Ullmark G, Nivbrant B. Migration pattern of a short femoral neck preserving stem. Clin Orthop Relat Res. 2006; 448: 73-8. doi: 10.1097/01.blo.0000224000.87517.4c

56. Sesselmann S, Hong YT, Schlemmer F, Hussnaetter I, Mueller LA, Forst R, et al. Radiostereometric migration measurement of an uncemented Cerafit® femoral stem: 26 patients followed for 10 years. Biomed Tech (Berl). 2018; 63(6): 657-63. doi: 10.1515/bmt-2016-0251

57. Simpson DJ, Kendrick BJL, Hughes M, Glyn-Jones S, Gill HS, Rushforth GF, et al. The migration patterns of two versions of the Furlong cementless femoral stem A randomised, controlled trial using radiostereometric analysis. J Bone Joint Surg Br. 2010; 92B(10): 1356-62. doi: 10.1302/0301-620X.92B10.24399

58. Søballe K, Toksvig-Larsen S, Gelineck J, Fruensgaard S, Hansen ES, Ryd L, et al. Migration of hydroxyapatite coated femoral prostheses. A roentgen stereophotogrammetric study. J Bone Joint Surg Br. 1993; 75(5): 681-7. doi: 10.1302/0301-620x.75b5.8397213

59. Steimer O, Adam F, Johann S, Pape D. Primary stability of cementless implanted hip stems made of titanium alloy with metaphyseal fixation. A prospective clinical Roentgen-Stereometry-Analysis (RSA) study. Z Orthop Ihre Grenzgeb. 2006; 144(6): 587-93. doi: 10.1055/s-2006-955188

60. Ström H, Kolstad K, Mallmin H, Sahlstedt B, Milbrink J. Comparison of the uncemented Cone and the cemented Bimetric hip prosthesis in young patients with osteoarthritis: An RSA, clinical and radiographic study. Acta Orthop. 2006; 77(1): 71-8. doi: 10.1080/17453670610045713

61. Ström H, Mallmin H, Milbrink J, Petrén-Mallmin M, Nivbrant B, Kolstad K. The Cone hip stem: A prospective study of 13 patients followed for 5 years with RSA. Acta Orthop Scand. 2003; 74(5): 525-30. doi: 10.1080/00016470310017901

62. Thien TM, Ahnfelt L, Eriksson M, Strömberg C, Kärrholm J. Immediate weight bearing after uncemented total hip arthroplasty with an anteverted stem: A prospective randomized comparison using radiostereometry. Acta Orthop. 2007; 78(6): 730-8. doi: 10.1080/17453670710014491

63. Thien TM, Thanner J, Kärrholm J. Fixation and bone remodeling around a low-modulus stem Seven-year follow-up of a randomized study with use of radiostereometry and dual-energy x-ray absorptiometer. J Arthroplasty. 2012; 27(1): 134-42.e1. doi: 10.1016/j.arth.2011.03.029

64. Turgeon TR, Hedden DR, Bohm ER, Burnell CD. Radiostereometric analysis and clinical outcomes of a novel reverse total hip system at two years. Bone Jt Open. 2023; 4(5): 385-92. doi: 10.1302/2633-1462.45.Bjo-2023-0018.R1

65. Turgeon TR, Righolt CH, Burnell CD, Gascoyne TC, Hedden DR, Bohm ER. Comparison of two hydroxyapatite-coated femoral components: A randomized clinical trial using radiostereometric analysis. Bone Joint J. 2023; 105-b(10): 1045-51. doi: 10.1302/0301-620x.105b10.Bjj-2023-0427.R1

66. Van Der Voort P, M.L DKN, Valstar ER, Kaptein BL, Fiocco M, R GHHN. Long-term migration of a cementless stem with different bioactive coatings. Data from a "prime" RSA study: lessons learned. Acta Orthop. 2020; 91(6): 660-8. doi: 10.1080/17453674.2020.1840021

67. van der Voort P, van Delft D, Valstar ER, Kaptein BL, Fiocco M, Nelissen RGHH. Migration behaviour of 2 clinically excellent cementless stems with different design rationales: 5-year follow-up of a randomised RSA-study. Hip Int. 2022; 32(6): 747-58. doi: 10.1177/1120700021995482

68. Weber E, Flivik C, Sundberg M, Flivik G. Migration pattern of a short uncemented stem with or without collar: A randomised RSA-study with 2 years follow-up. Hip Int. 2021; 31(4): 500-6. doi: 10.1177/1120700019888471

69. Weber E, Sundberg M, Flivik G. Design modifications of the uncemented Furlong hip stem result in minor early subsidence but do not affect further stability: A randomized controlled RSA study with 5-year follow-up. Acta Orthop. 2014; 85(6): 556-61. doi: 10.3109/17453674.2014.958810

70. Wolf O, Mattsson P, Milbrink J, Larsson S, Mallmin H. Periprosthetic bone mineral density and fixation of the uncemented CLS stem related to different weight bearing regimes: A randomized study using DXA and RSA in 38 patients followed for 5 years. Acta Orthop. 2010; 81(3): 286-91. doi: 10.3109/17453674.2010.487238

71. Wykman A, Lundberg A. Subsidence of porous coated noncemented femoral components in total hip arthroplasty. A roentgen stereophotogrammetric analysis. J Arthroplasty. 1992; 7(2): 197-200. doi: 10.1016/0883-5403(92)90017-k

72. Wykman A, Selvik G, Goldie I. Subsidence of the femoral component in the noncemented total hip. A roentgen stereophotogrammetric analysis. Acta Orthop Scand. 1988; 59(6): 635-7. doi: 10.3109/17453678809149414

73. Zampelis V, Flivik G, Kesteris U. No effect of femoral canal jet-lavage on the stability of cementless stems in primary hip arthroplasty: A randomised RSA study with 6 years follow-up. Hip Int. 2020; 30(4): 417-22. doi: 10.1177/1120700019843123

Appendix C: Extra Tables and Figures

Table C1. Pooled mean and confidence interval of the 2-year follow-up subsidence and retroversion per stem subgroup.

	Subsidence (mm)			*Retroversion (degrees)*		
	mean	***CI***	***cohorts***	***mean***	***CI***	***cohorts***
Coating						
HA-coated stems	0.26	0.13 to 0.40	54	0.51	0.22 to 0.80	42

Porous coated stems	0.56	0.29 to 0.82	30	1.32	0.67 to 1.97	20
Uncoated stems	0.37	0.03 to 0.70	11	0.69	0.36 to 1.02	11
Stem type						
Flat-taper stems	0.29	0.06 to 0.52	16	0.68	0.09 to 1.27	9
Quadrangular taper stems	0.43	0.07 to 0.80	15	0.81	-0.17 to 1.79	10
Fit-and-Fill stems	0.41	0.24 to 0.59	49	0.85	0.49 to 1.21	42
Cylindrical stems	-0.01	-0.57 to 0.56	2	1.26	-0.41 to 2.93	2
Conical stems	0.47	-0.36 to 1.31	3	0.81	-1.60 to 3.22	1
Ultra-short neck-preserving stems	0.12	-0.03 to 0.27	9	0.50	0.32 to 0.68	8
Other stems	0.08	-0.15 to 0.31	5	0.14	-0.90 to 1.18	3
Inclusion period						
1980s	0.53	0.12 to 0.94	8	2.60	1.64 to 3.56	1
1990s	0.05	-0.10 to 0.19	10	0.51	0.31 to 0.71	7
2000s	0.36	0.16 to 0.55	37	0.48	0.21 to 0.75	34
2010s	0.37	0.20 to 0.54	44	0.81	0.45 to 1.16	33
Surgical approach						
Direct anterior approach	1.04	0.53 to 1.55	3	1.52	1.08 to 1.95	3
Anterolateral approach	0.11	-0.03 to 0.25	15	0.51	0.33 to 0.68	15
Direct lateral approach	0.36	0.08 to 0.64	20	0.20	-0.23 to 0.64	11
Posterior approach	0.32	0.16 to 0.47	39	1.03	0.54 to 1.53	32
Mixed approach	0.18	-0.06 to 0.42	15	0.95	0.23 to 1.67	9
RSA technique						
Marker-based RSA	0.29	0.15 to 0.42	57	0.62	0.35 to 0.89	40
Model-based RSA	0.37	0.19 to 0.54	40	0.81	0.43 to 1.18	33
Randomization						
Randomized	0.19	0.09 to 0.29	68	0.69	0.45 to 0.93	53
Non-randomized	0.38	0.19 to 0.57	31	0.78	0.24 to 1.33	22
Weight-bearing						
Weight-bearing studies	0.20	0.09 to 0.30	62	0.76	0.43 to 1.09	46
Non-weight-bearing studies	0.58	0.26 to 0.91	18	1.01	0.38 to 1.63	15
baseline measurement						
Overall mean incl. delayed baseline	0.28	0.18 to 0.38	105	0.66	0.44 to 0.88	79
Overall mean excl. delayed baseline	0.32	0.21 to 0.43	99	0.70	0.48 to 0.93	75
Mean of delayed baseline studies	0.01	-0.08 to 0.10	6	0.04	-0.68 to 0.75	4

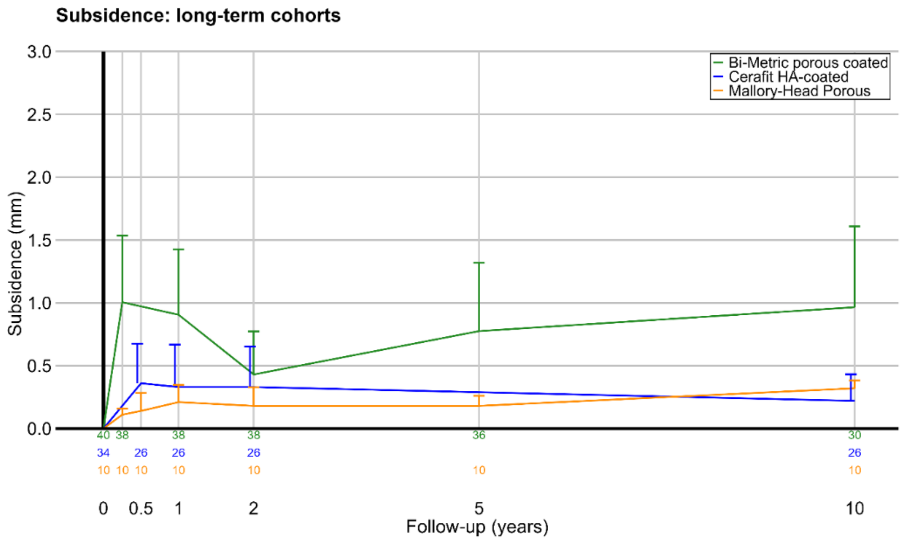

Figure C1. Long-term mean subsidence pattern of Bi-Metric porous coated stems, Cerafit HA-coated stems, and Mallory-Head Porous stems (with mixed coating). The error bars represent the upper limit of the 95% confidence intervals, and the numbers below the graph indicate the number of hips per group at each follow-up.

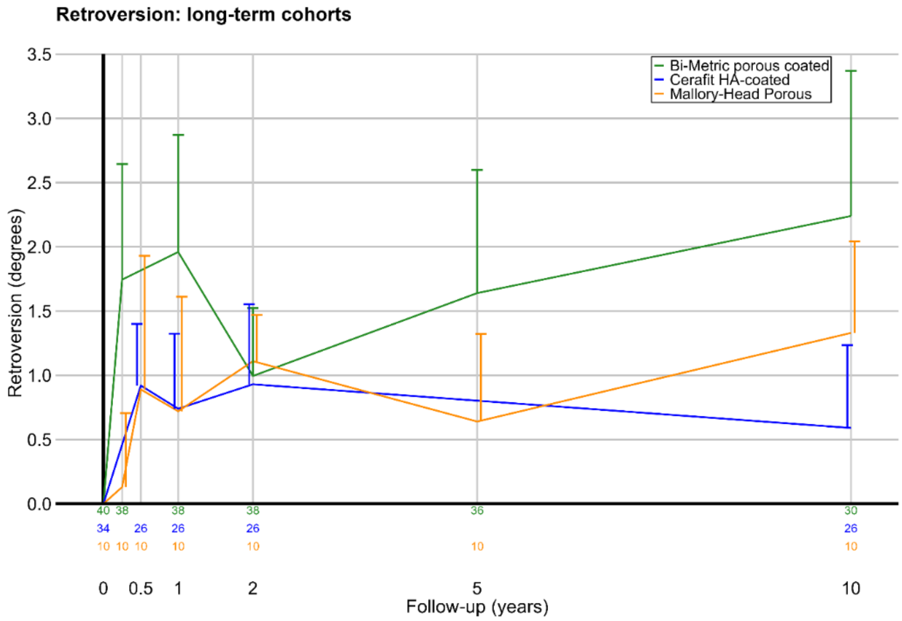

Figure C2. Long-term mean retroversion patterns of Bi-Metric porous coated stems, Cerafit HA-coated stems, and Mallory-Head Porous stems (with mixed coating). The error bars represent the upper limit of the 95% confidence intervals, and the numbers below the graph indicate the number of hips per group at each follow-up.

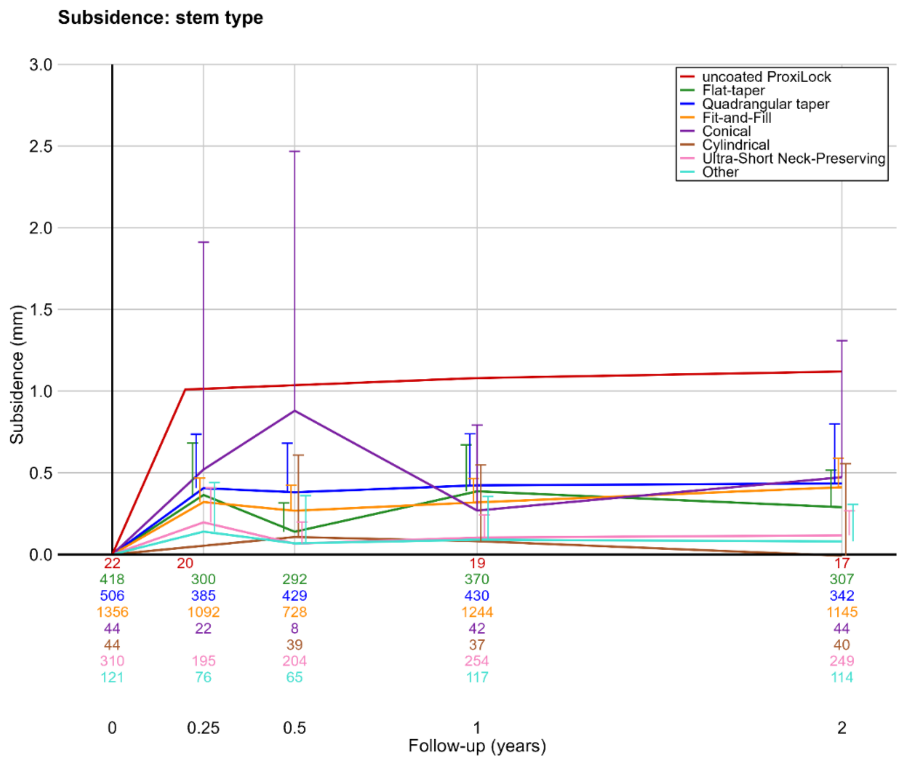

Figure C3. Pooled mean subsidence during the first 2 years per stem type, with the known failure uncoated ProxiLock for reference (8). The error bars represent the upper 95% confidence intervals, and the numbers below the graph indicate the number of hips per group at each follow-up.

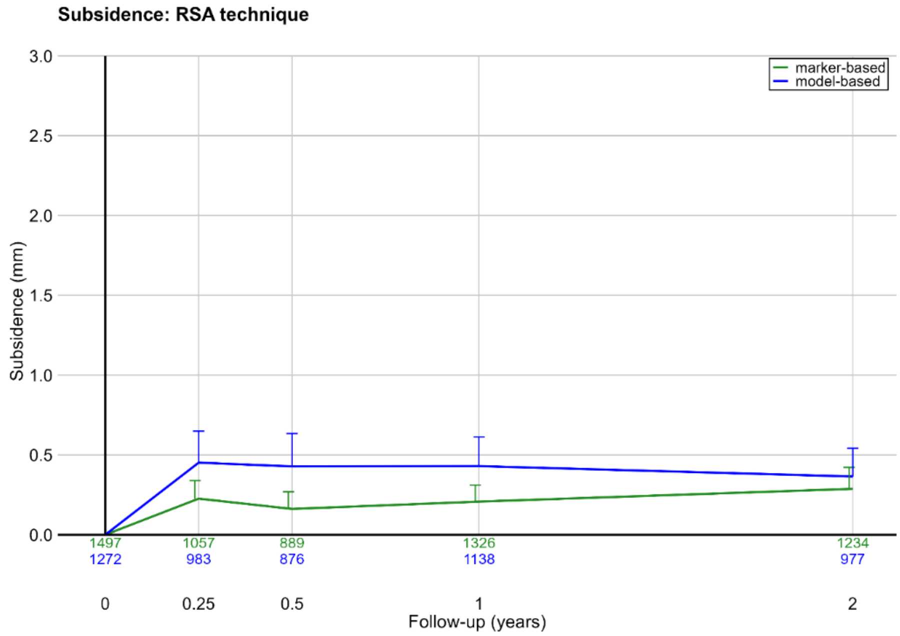

Figure C4. Pooled mean subsidence during the first 2 years for marker- and model-based RSA studies. The error bars represent the upper 95% confidence intervals, and the numbers below the graph indicate the number of hips per group at each follow-up.

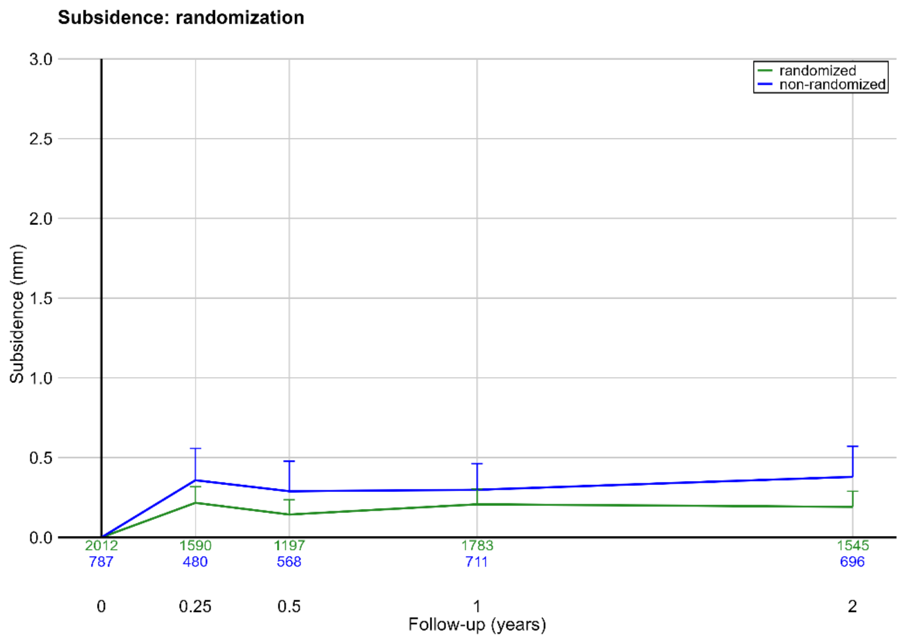

Figure C5. Pooled mean subsidence during the first 2 years for randomized and non-randomized studies. The error bars represent the upper 95% confidence intervals, and the numbers below the graph indicate the number of hips per group at each follow-up.

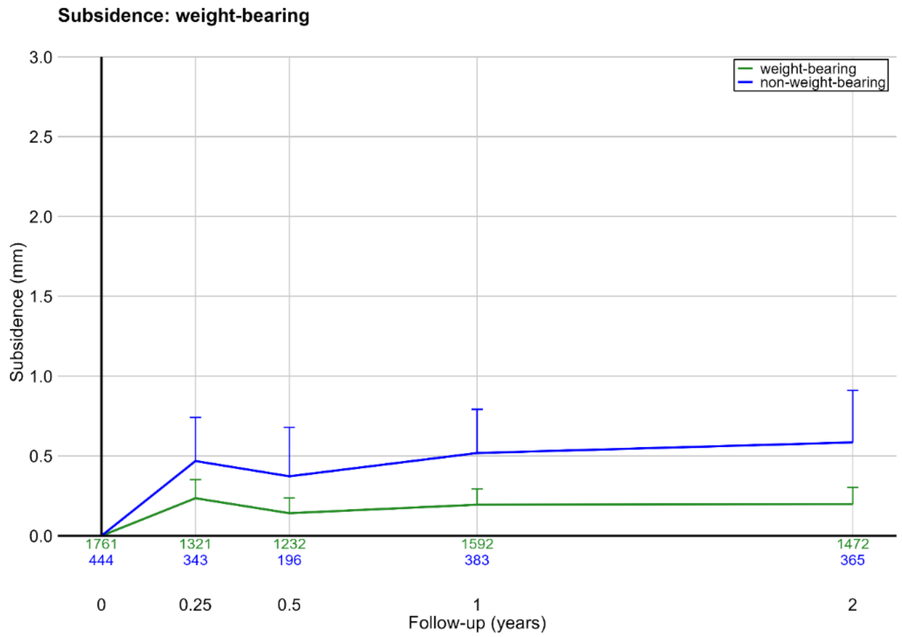

Figure C6. Pooled mean subsidence during the first 2 years for weight-bearing and non-weight-bearing cohorts. The error bars represent the upper 95% confidence intervals, and the numbers below the graph indicate the number of hips per group at each follow-up.

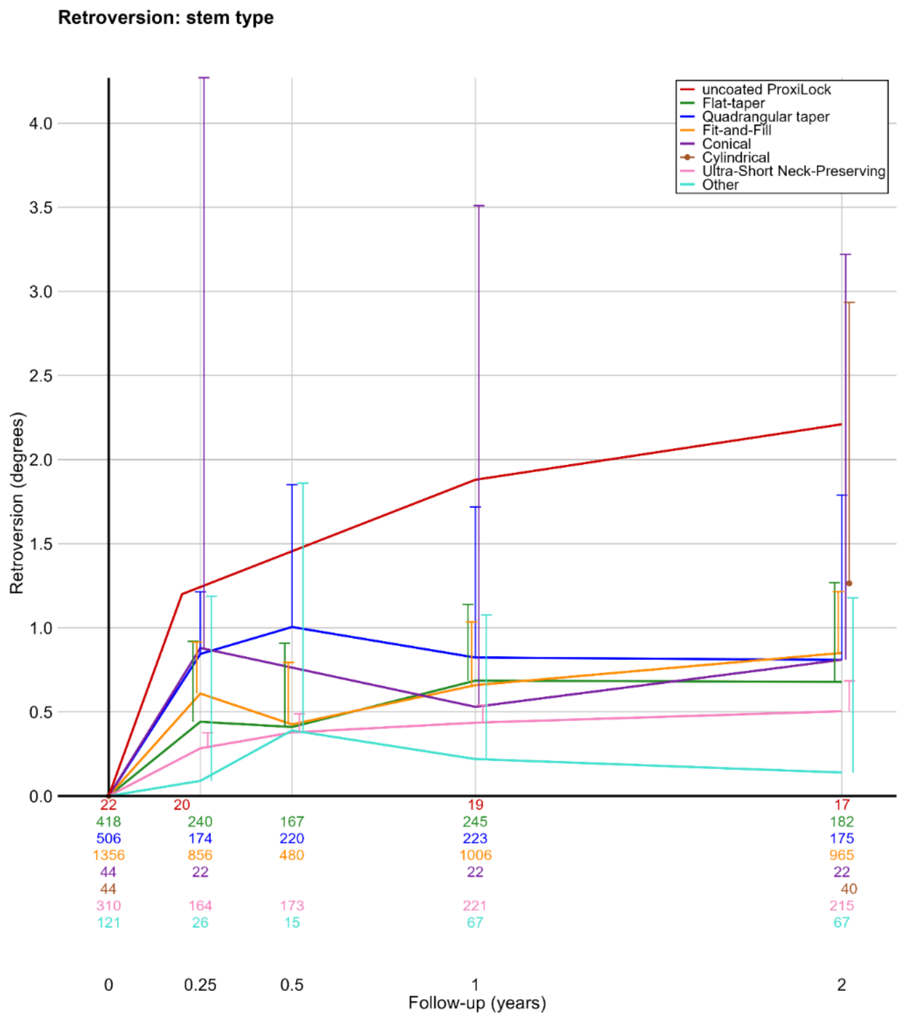

Figure C7. Pooled mean retroversion during the first 2 years per stem type, with the known failure uncoated ProxiLock for reference (8). For the Cylindrical stems there was no retroversion pattern available, so just the 2-year retroversion result is presented. The error bars represent the upper 95% confidence intervals, and the numbers below the graph indicate the number of hips per group at each follow-up.

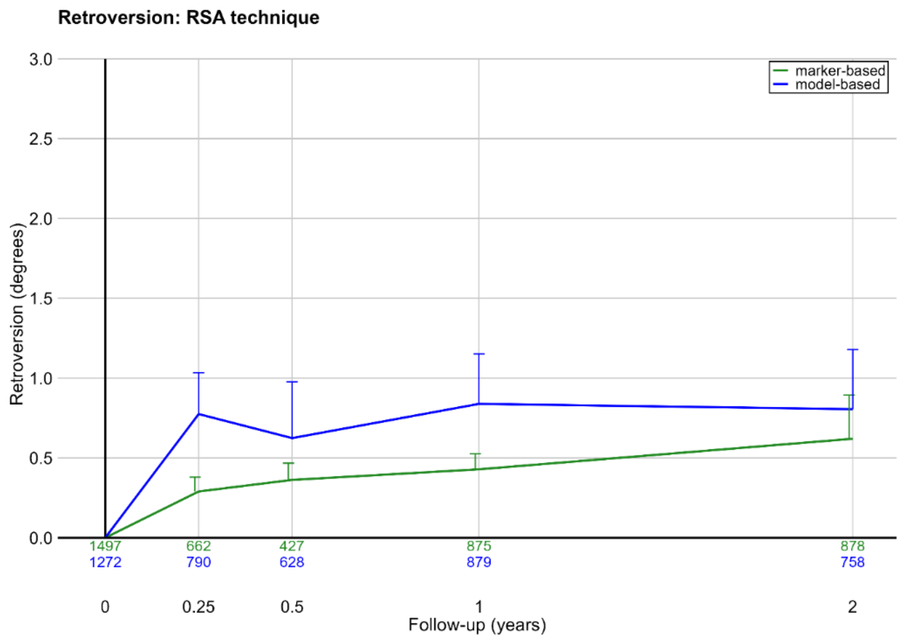

Figure C8. Pooled mean retroversion during the first 2 years for marker- and model-based RSA studies. The error bars represent the upper 95% confidence intervals, and the numbers below the graph indicate the number of hips per group at each follow-up.

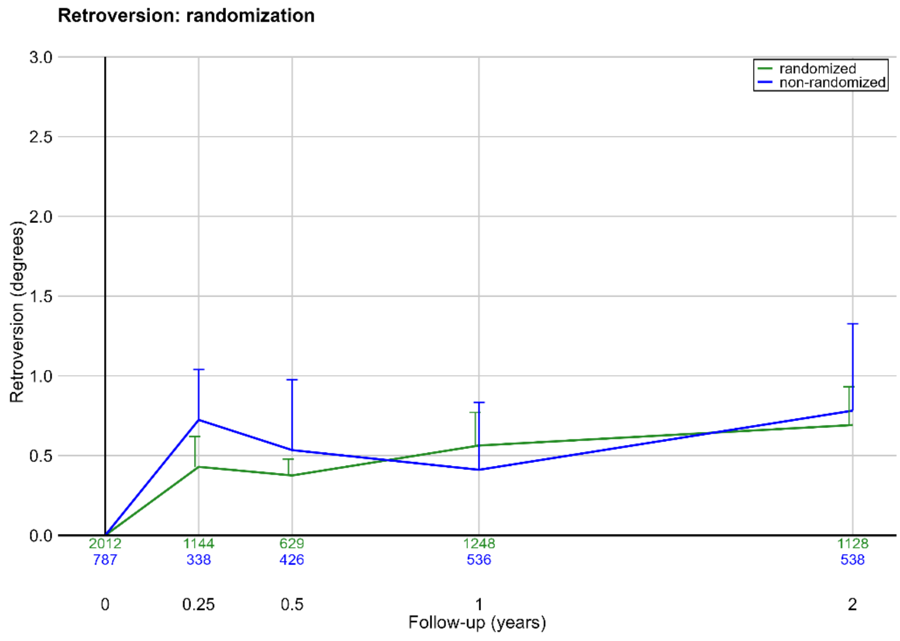

Figure C9. Pooled mean retroversion during the first 2 years for randomized and non-randomized studies. The error bars represent the upper 95% confidence intervals, and the numbers below the graph indicate the number of hips per group at each follow-up.

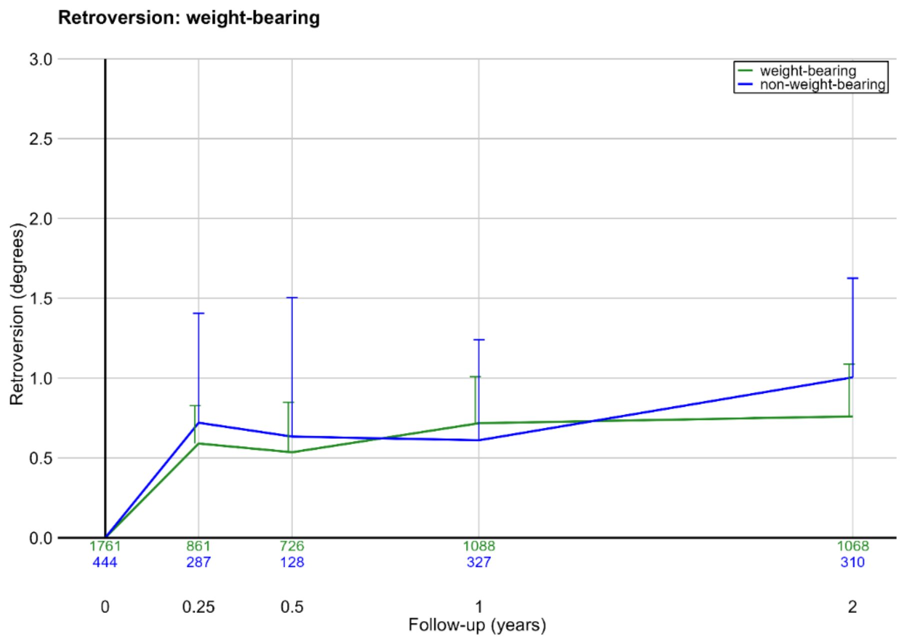

Figure C10. Pooled mean retroversion during the first 2 years for weight-bearing and non-weight-bearing cohorts. The error bars represent the upper 95% confidence intervals, and the numbers below the graph indicate the number of hips per group at each follow-up.

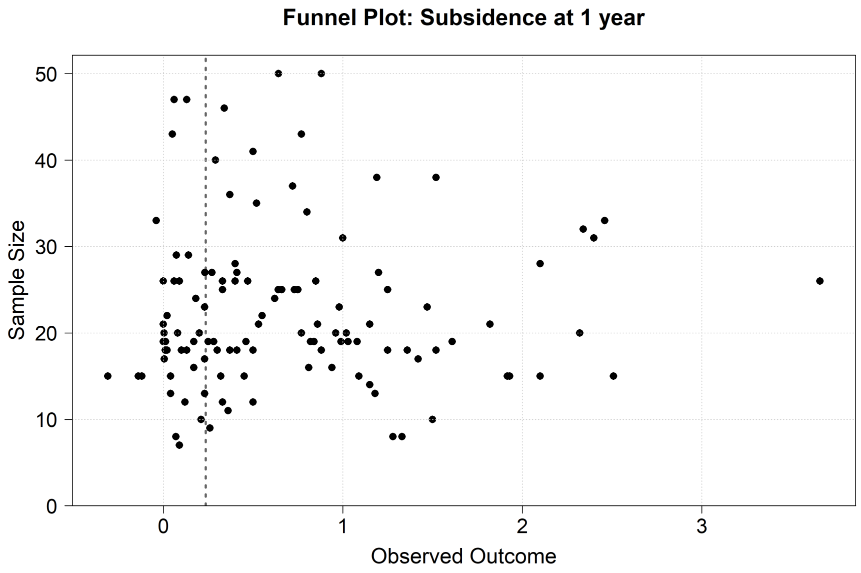

Figure C11. Funnel plot with subsidence on the x-axis and sample size on the y-axis.

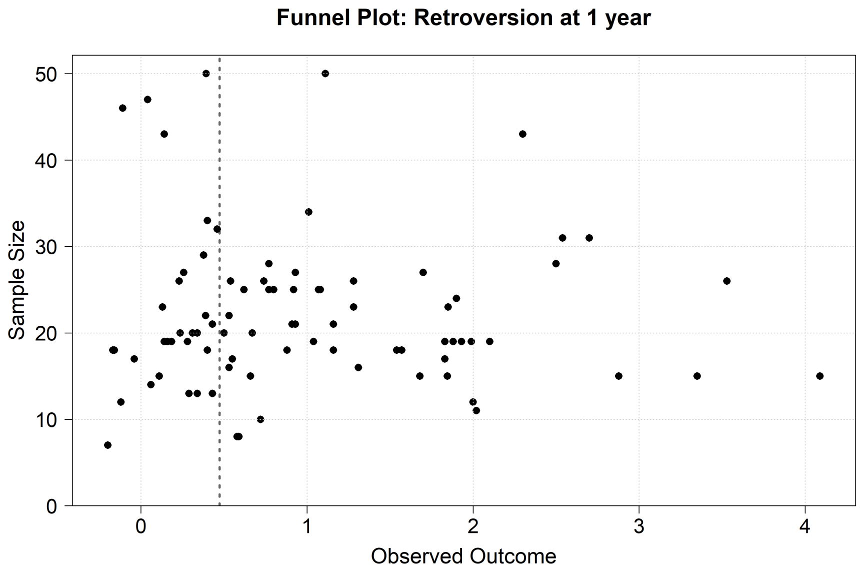

Figure C12. Funnel plot with subsidence on the x-axis and sample size on the y-axis.

Forest Plot – Subsidence at 3 Months

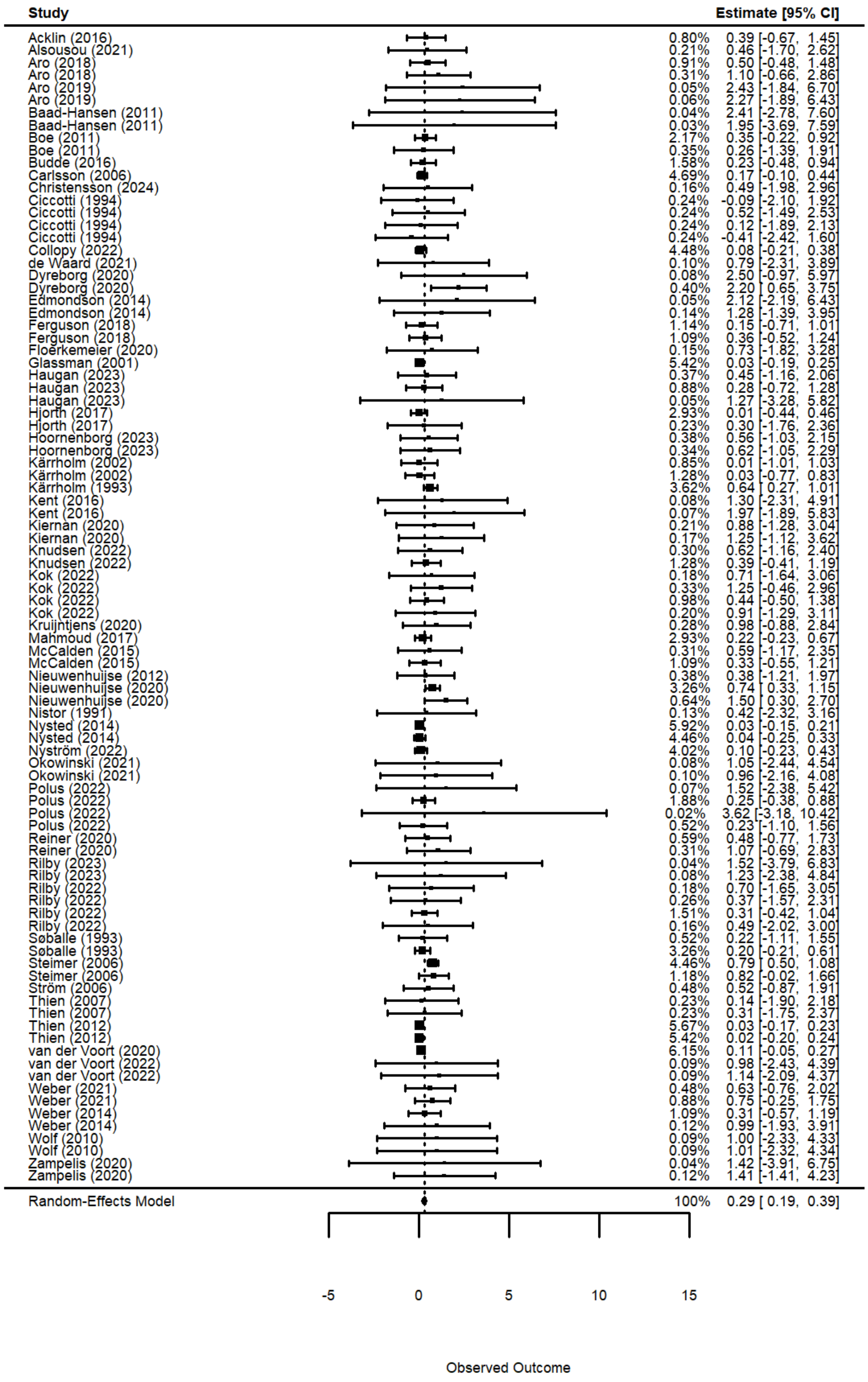

Figure C13. Forest plot of subsidence at 3 months.

Forest Plot – Subsidence at 2 Years

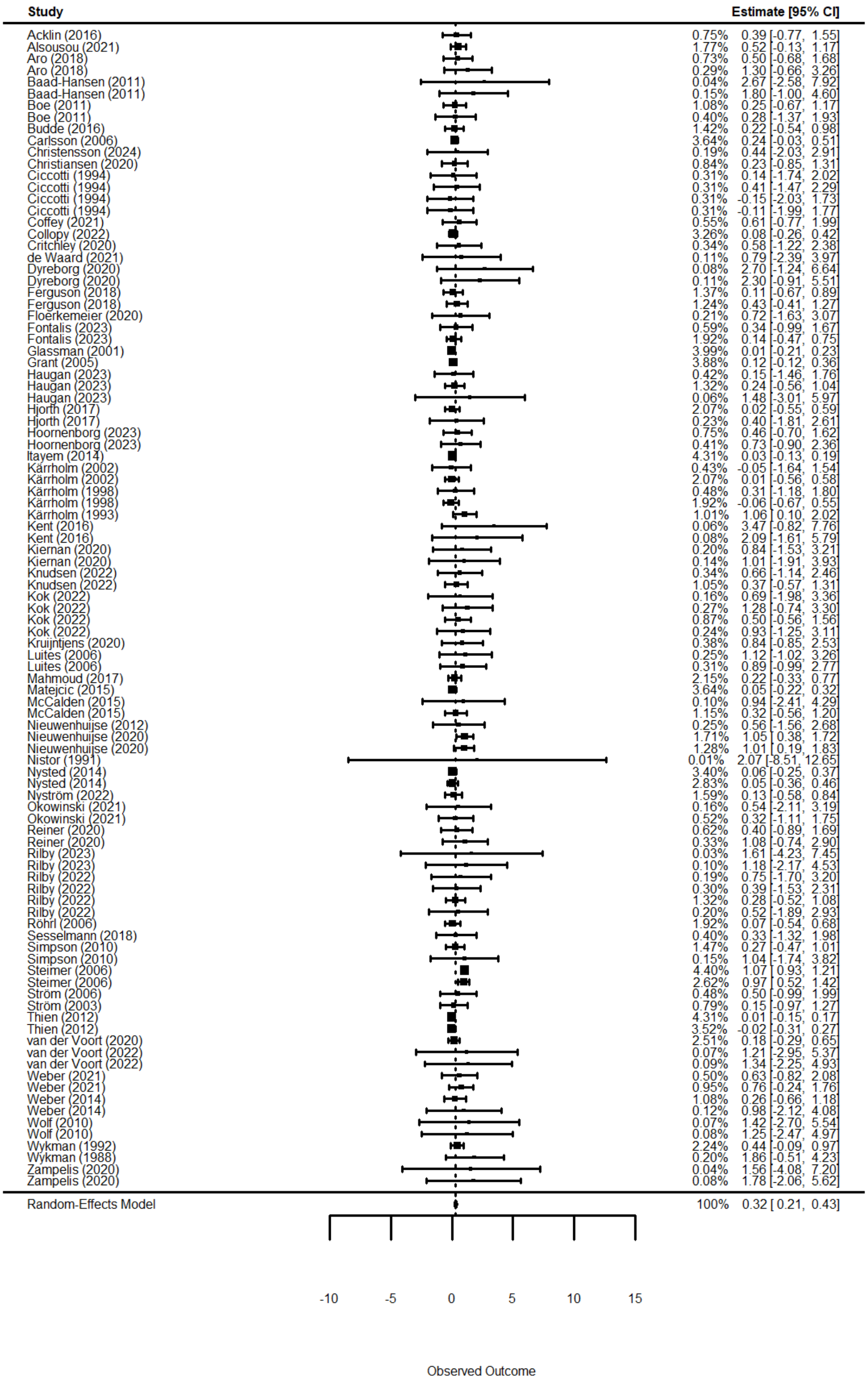

Figure C14. Forest plot of subsidence at 2 years.

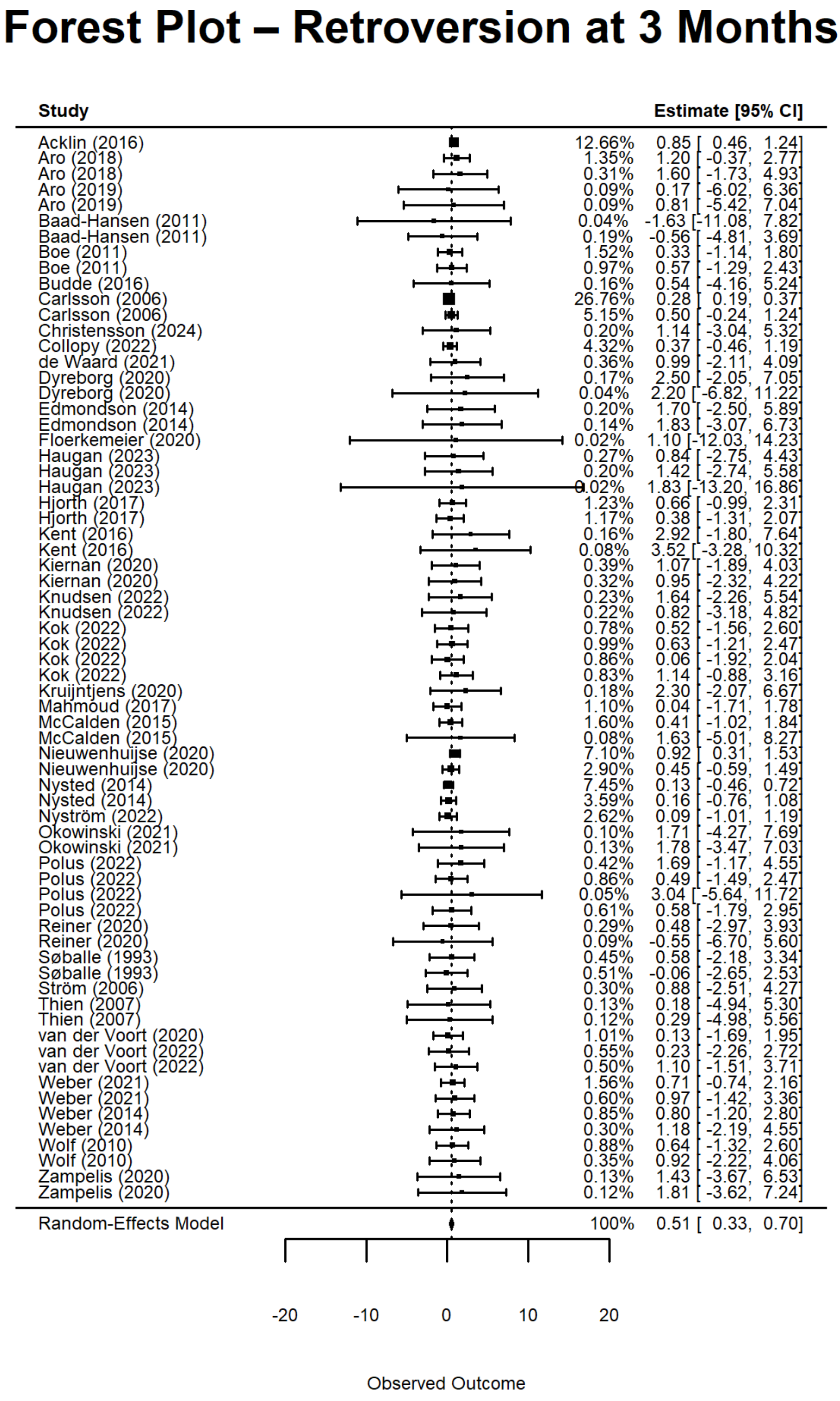

Figure C15. Forest plot of retroversion at 3 months.

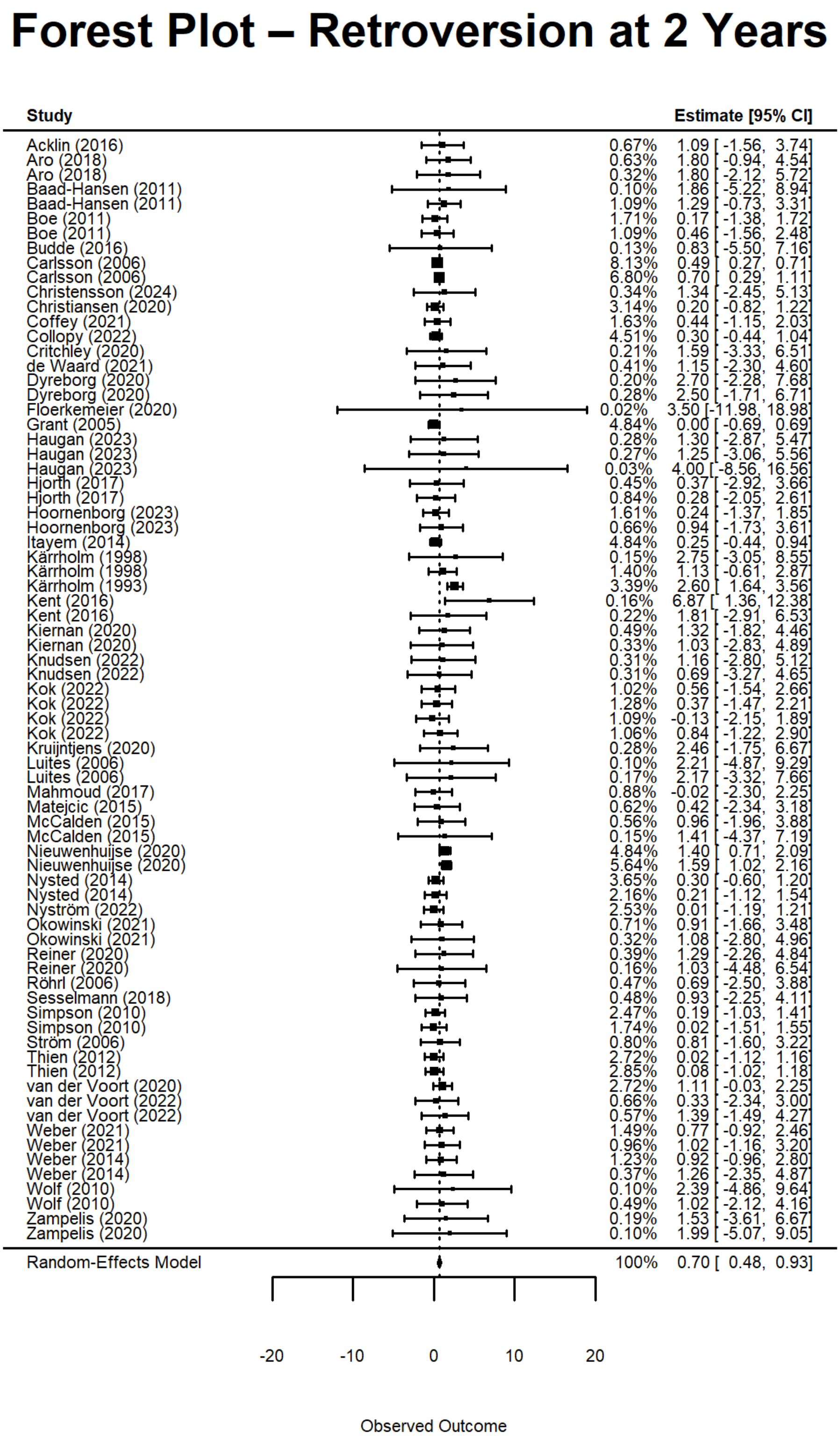

Figure C16. Forest plot of retroversion at 2 years.
